# Evaluating the state of the science for adeno-associated virus integration: An integrated perspective

**DOI:** 10.1016/j.ymthe.2022.06.004

**Published:** 2022-06-10

**Authors:** Denise E. Sabatino, Frederic D. Bushman, Randy J. Chandler, Ronald G. Crystal, Beverly L. Davidson, Ricardo Dolmetsch, Kevin C. Eggan, Guangping Gao, Irene Gil-Farina, Mark A. Kay, Douglas M. McCarty, Eugenio Montini, Adora Ndu, Jing Yuan, The American Society of Gene and Cell Therapy (ASGCT) Working Group on AAV Integration

**Affiliations:** 1 The Raymond G. Perelman Center for Cellular and Molecular Therapeutics, The Children’s Hospital of Philadelphia, Philadelphia, PA, USA; 2 Division of Hematology, Department of Pediatrics, Perelman School of Medicine, University of Pennsylvania, Philadelphia, PA, USA; 3 Department of Microbiology, Perelman School of Medicine, University of Pennsylvania, Philadelphia, PA, USA; 4 National Human Genome Research Institute, NIH, Bethesda, MD, USA; 5 Department of Genetic Medicine, Weill Medical College of Cornell University, New York, NY, USA; 6 Department of Pathology and Laboratory Medicine, Perelman School of Medicine, University of Pennsylvania, Philadelphia, PA, USA; 7 uniQure N.V., Amsterdam, the Netherlands; 8 BioMarin Pharmaceutical Inc, San Rafael, CA, USA; 9 Horae Gene Therapy Center, University of Massachusetts Medical School, Worcester, MA, USA; 10 ProtaGene CGT GmbH, Heidelberg, Germany; 11 Departments of Pediatrics and Genetics, Stanford University, Stanford, CA, USA; 12 NeuroGT, Inc., Durham, NC, USA; 13 San Raffaele Telethon Institute for Gene Therapy, IRCCS San Raffaele Scientific Institute, Milan, Italy; 14 BridgeBio Pharma, Inc., Palo Alto, CA, USA; 15 Drug Safety Research and Development, Pfizer Inc., Cambridge, MA, USA

**Keywords:** AAV, genotoxicity, oncogenicity risk, genome integration, insertional mutagenesis, recombinant AAV (rAAV), clonal expansion, hepatocellular carcinoma (HCC), next-generation sequencing, bioethics

## Abstract

On August 18, 2021, the American Society of Gene and Cell Therapy (ASGCT) hosted a virtual roundtable on adeno-associated virus (AAV) integration, featuring leading experts in preclinical and clinical AAV gene therapy, to further contextualize and understand this phenomenon. Recombinant AAV (rAAV) vectors are used to develop therapies for many conditions given their ability to transduce multiple cell types, resulting in long-term expression of transgenes. Although most rAAV DNA typically remains episomal, some rAAV DNA becomes integrated into genomic DNA at a low frequency, and rAAV insertional mutagenesis has been shown to lead to tumorigenesis in neonatal mice. Currently, the risk of rAAV-mediated oncogenesis in humans is theoretical because no confirmed genotoxic events have been reported to date. However, because insertional mutagenesis has been reported in a small number of murine studies, there is a need to characterize this genotoxicity to inform research, regulatory needs, and patient care. The purpose of this white paper is to review the evidence of rAAV-related host genome integration in animal models and possible risks of insertional mutagenesis in patients. In addition, technical considerations, regulatory guidance, and bioethics are discussed.

## Graphical abstract

**Figure:**
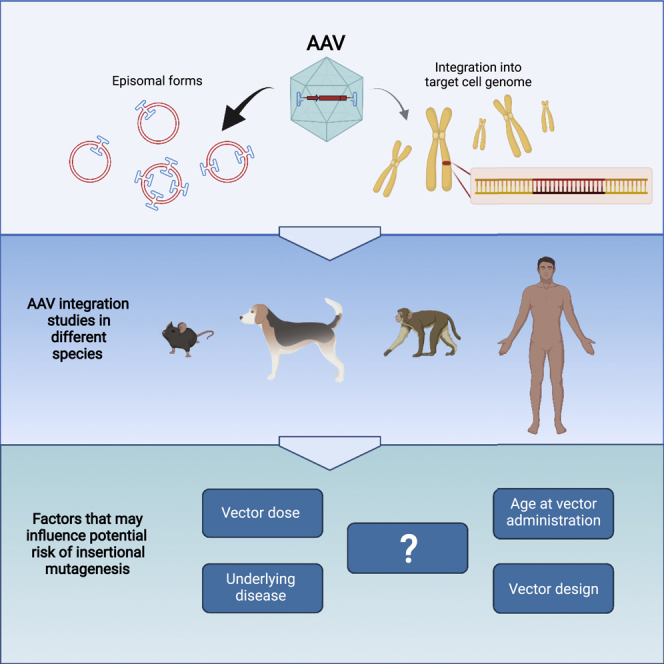

## Teaser

While recombinant AAV DNA remains primarily episomal, it can integrate into the target cell genome. This review discusses the current understanding of AAV integration and the potential risk of genotoxicity. We discuss the factors that may influence the risk of insertional mutagenesis, technical considerations, and regulatory guidance.

## Introduction

A goal of the American Society of Gene and Cell Therapy (ASGCT) is to serve as a catalyst for bringing together relevant stakeholders in the field to discuss key issues. To this end, the ASGCT hosted a virtual roundtable in August 2021 on the integration of DNA of the widely used gene delivery vectors derived from recombinant adeno-associated virus (rAAV).^1^ The overall goal was to inform further research, gain insight into regulatory needs, and provide guidance on informed consent and patient care. The session featured leading stakeholders from the academic, pharmaceutical, and biotechnology sectors. There are several ongoing regulatory and policy discussions about adeno-associated virus (AAV) vector-based gene therapy products, including the September 2021 US Food and Drug Administration (FDA) Cellular, Tissue, and Gene Therapies Advisory Committee (CTGTAC) meeting,^2^ the 2021 ASGCT Policy Summit,^3^ and the November 2021 ASGCT-FDA Liaison Meeting.^4^ Key insights from these informative meetings are presented later in this paper.

Viral vectors take advantage of the innate ability of viruses to deliver genetic material into cells. AAV, the topic of this white paper, is generally regarded as non-pathogenic; in the wild-type (WT) state AAV is not associated with known pathologies. While integrating retroviral and lentiviral vectors serve as gene delivery vehicles driving long-term expression in both dividing and non-dividing cells,^5^^,^^6^ rAAV is commonly used for *in vivo* therapeutic delivery because it provides sustained transgene expression in a variety of quiescent cell types. Given the broad use of rAAV vectors for gene therapy, understanding the state of rAAV DNA in cells months to years after gene delivery becomes important for safe and effective implementation.

There are 13 AAV serotypes (AAV1 to AAV13) and many additional variants that are derived from humans, other primates, and other mammalian species that have been adapted as vectors.^7^^,^^8^ In addition, there are a number of chimeric capsids that have been selected from DNA libraries or rationally designed, which have enhanced properties and have entered clinical trials.^7^^,^^9^ AAV serotypes differ based on the composition of the capsid protein, and as a result target different tissues, known as tropism.^7^^,^^10^ For example, some AAVs exhibit preferential tropism for muscle,^11^ others target liver,^12^ while others target the central nervous system (CNS) and peripheral nervous system (PNS) after intravenous delivery.^13^^,^^14^

WT AAV has a single-stranded 4.7-kb DNA genome that is flanked by inverted terminal repeat sequences (ITRs). The genome encodes two genes: the *rep* gene, which is required for replication and packaging of the DNA, and the *cap* gene, which encodes the proteins that assemble into the viral capsid. In rAAV, the *rep* and *cap* genes are removed and replaced with the transgene expression cassette; thus, the only remaining viral genome sequences are the ITRs that are essential for packaging of the vector genome.^7^ rAAV vector genome processing relies on host cellular machinery such as DNA repair systems in order to facilitate stable transduction.^15^ Following AAV vector transduction, single stranded rAAV vector genomes are used as templates to form double-stranded (ds) linear rAAV monomers.^16^ These go on to form ds circular monomers and DNA concatemers directed by host DNA polymerases and DNA repair pathways.^17^^,^^18^ WT AAV can be maintained as an episome or integrate into the target cell genome mediated by *Rep* occurring preferentially at a locus on chromosome 19 in humans and also at other chromosome locations.^19^, ^20^, ^21^

Unlike WT AAV, rAAV DNA lacks the Rep-mediated active integration and primarily remains in a circular concatemeric episomal form.^22^ However, AAV vectors integrate at low frequency into the target cell genome, likely mediated by host cell DNA-modifying enzymes.^17^^,^^22^^,^^23^ Thus, there is a risk that rAAV genome integration could result in genotoxicity. Animal data have provided evidence that rAAV vectors delivered in mice may contribute to the formation of hepatocellular carcinoma (HCC), but the relevance of these findings to the risks associated with the use of rAAVs in humans remains unknown.^24^, ^25^, ^26^, ^27^

Three gene therapy drugs based on rAAV vectors have been approved by the FDA and European Medicines Agency (EMA) for the treatment of diseases with limited therapeutic options: Glybera (alipogene tiparvovec) for the treatment of familial lipoprotein lipase deficiency (LPLD),^28^ LUXTURNA (voretigene neparvovec-rzyl) for the treatment of vision loss or complete blindness in certain patients,^29^^,^^30^ and Zolgensma (onasemnogene abeparvovec-xioi) for the treatment of spinal muscular atrophy (SMA) in certain pediatric patients.^31^^,^^32^ In addition, rAAV-based vectors are being used in clinical trials to treat many diseases, including muscular diseases (Duchenne muscular dystrophy, X-linked myotubular myopathy), hemophilia, inherited metabolic disorders (ornithine transcarbamylase deficiency, methylmalonic acidemia), neurological diseases (Parkinson’s disease, Batten’s disease), and ocular diseases (retinitis pigmentosa, choroideremia, age-related macular degeneration).^15^^,^^33^^,^^34^ To date, no confirmed genotoxic events in humans have been reported from the use of rAAV vectors.

This white paper will review the evidence of AAV vector-related host genome integration in animal models and risk of insertional mutagenesis in patients. In addition, an overview of bioethics, expert opinions, and regulatory guidelines is presented.

## AAV vector-related host genome integration in animal models

Early studies of AAV integration in the late 1990s and early 2000s were performed in both *in vitro* and *in vivo* models. The studies in cell lines^35^^,^^36^ and in mouse models^37^ observed near-random integration that favored actively transcribed regions of the genome. In a study that utilized a partial hepatectomy mouse model, <10% of the AAV genomes were estimated to be integrated.^38^ It is notable that the methods used in these studies relied on Southern blot analysis and construction of rAAV vector-cellular DNA junction fragment libraries to characterize the integrated AAV forms. Further studies that characterized the integration sites in more detail found that the majority of integration events were in intragenic locations, in transcriptionally active regions, near CpG islands, or near transcription start sites.^17^^,^^39^^,^^40^ In addition, studies suggested that rAAV integration was dependent on ds DNA breaks in the genome.^41^

### Evidence of rAAV genotoxicity from neonatal mice

The first association between rAAV and tumorigenesis was made in studies designed to test the efficacy of rAAV gene delivery in a murine model of mucopolysaccharidosis type VII (MPSVII) (Table 1).^42^ MPSVII is a lysosomal storage disease caused by a deficiency of beta-glucuronidase (GUSB).^43^ MPSVII mice were intravenously injected with an AAV2 vector containing a cytomegalovirus enhancer/chicken beta-actin (CAG) promoter and a human beta-glucuronidase (GUSB) cDNA at the newborn stage (postnatal day 2) with a dose of 5 × 10^9^ or 8 × 10^10^ vector genomes (vg)/pup (∼4 × 10^12^vg/kg or ∼7 × 10^13^ vg/kg). A single rAAV treatment in the neonatal period of MPSVII mice resulted in long-term transgene expression, GUSB activity, and phenotypic correction.^44^^,^^45^ A number of long-lived rAAV-treated MPSVII mice that were over a year old developed HCC and angiosarcomas.^42^^,^^46^ The tumor samples were negative for GUSB activity and the rAAV genome could not be detected in the tumor genome by polymerase chain reaction (PCR) using primers specific for the transgene GUSB cDNA, thus suggesting that the tumors were probably not caused by insertional mutagenesis. However, a larger follow-up study in newborn MPSVII with intravenous injection of the same AAV2 vector resulted in 30%–60% of both normal and MPSVII mice developing HCC at about 13 months of age.^46^ In contrast to the first study,^42^ this study detected rAAV integrations in the tumors, which suggested that the inability to detect AAV integrations in the tumors in the original study may have been due to technical limitations of the PCR approach.

**Table 1:** Summary of mouse studies that observed or investigated HCC formation after rAAV gene delivery

Publication	AAV serotype	Genome configuration	Enhancer/promoter (∗promoters only)	Transgene	Route of delivery	Dose	Time of treatment	Loci with integration(s) associated with HCC
Donsante 2001^42^ Donsante 2007^46^	2	SS	CMV/CBA	*GUSB*	superficial temporal vein	1 × 10^14^ vg/kg	neonatal	*Rian*
Bell 2005^47^	1,2,5,7,8,9, others	SS	Alb, CBA, SV40, TBG, Z12∗	*A1AT*, *EGFP*, *Epo*, *FIX*, *LacZ*, *LDLR*, *TF1*	portal vein tail vein	3 × 10^9^-1 × 10^12^ vg/mouse	6–8 weeks	none
Bell 2006^48^ Zhong 2013^49^	2,7,8,9	SS	AMPB/TBG	*Oct*, *LacZ*	portal vein	3 × 10^9^-1 × 10^12^ vg/mouse	9–11 weeks	*Tax1bp1*, *Rian*
Reiss 2011^50^	1/2	SS	CMV/CBA	*MOCS1*	intrahepatic	1–3 × 10^9^ vg/pup	neonatal	none
Embury 2008^51^	2	SS	CBA∗ (WPRE)	*mPah*	portal vein	3.7 × 10^9^-7.3 × 10^13^ vg/mouse	10–14 weeks	none
Li 2011^52^	2	SS	APOE/hAAT	*FIX*	portal vein	5 × 10^12^-1 × 10^14^ vg/kg	4 weeks	none
Wang 2012^53^	2	SS	CMV/CBA	*Homology Arms to Rian*	temporal vein	3 × 10^9^ vg/pup	neonatal	*Rian*
Rosas 2012^54^	Rh74	SC	CMV, CBA∗	*GFP*, *null*	tail vein	5 × 10^10^ vg/mouse	8–12 weeks	*Hras1*, *Sos1*, *Fgf3*
Kao 2013^55^	8	SS	APOE/hAAT	*FIX-WT*, *FIX-Triple*, *FIX-Padua*	tail vein	1.6 × 10^12^-2 × 10^10^ vg/mouse	adult	none
Chandler 2015^56^	2,8,9	SS	AMPB/TBG CMV/CBA APOE/hAAT	*hMUT*, *mMut*, *GFP*	intrahepatic	7 × 10^12^-1 × 10^14^ vg/kg	neonatal	*Rian*
Walia 2015^57^	9	SS	CMV/CMV	*HexB*, *LacZ*	superficial temporal vein	2.5 × 10^14^ vg/kg	neonatal	*Rian*
Kang 2019^58^	9	SS	TBG	*G6PC*, *ZFN*	tail vein	4.8 × 10^12^-1.3 × 10^13^ vg/kg	adult	*Rian*
Dalwadi 2021^59^	DJ	SS	CMV/CAG	*Homology Arms to Rian*, *tdTomato*	superficial temporal vein	3 × 10^10^ vg/pup 8 × 10^11^ vg/adult	neonatal adults	*Rian*
Li 2021^60^	9	SS	CMV/CBA	*GALC*	intracranial or intrathecal	2.7 × 10^10^ vg/pup	neonatal	*Rian*, *FoxP1*, *Tusc3*, *Magi1*, *Kcnh1*, *Akap13*

HCC, hepatic carcinoma; rAAV, recombinant adeno-associated virus; SS, single stranded; SC, self-complementary; CMV, cytomegalovirus; CBA, chicken beta-actin; Alb, albumin; TBG, thyroxin binding globulin; WPRE, woodchuck hepatitis virus post-transcriptional regulatory element; TBG, thyroxin binding globulin; APOE, apolipoprotein E; hAAT, human alpha-1 antitrypsin; CAG, cytomegalovirus enhancer/chicken beta-actin promoter.

Adapted from Chandler, 2017.^25^

The majority of these AAV integrations in the tumors were in the *Rian* (RNA imprinted and accumulated in nucleus) locus, which caused an upregulation of genes in and proximal to the *Rian* locus. *Rian* is a long-noncoding RNA that plays a role in epithelial to mesenchymal transition, negative regulation of Notch signaling pathway, and negative regulation of hepatic stellate cell activation. Mutation of the *Rian* locus has been shown to cause HCC in mice.^53^^,^^61^, ^62^, ^63^ *Rian* is orthologous to the human long non-coding RNA *MEG8*. Interestingly, increased expression of *MEG8* has been correlated with the poor prognosis of HCC patients.^64^ These observations led to the initial hypothesis that rAAV vector integrations in *Rian* dysregulated gene expression and contributed to tumorigenesis.

Since this initial observation, additional studies have observed HCC after rAAV delivery to mice. A study designed to determine the risk of tumorigenesis after AAV treatment of juvenile/adult mice with ornithine transcarbamylase (OTC) deficiency, which have a high incidence of liver tumors caused by the underlying disease, did not find an increase in tumor formation in the AAV-treated OTC mice.^48^ However, this study did report increased tumor formation in WT mice treated with a rAAV that expressed a LacZ reporter gene, although significance was unclear after statistical correction for multiple comparisons. A later AAV integration study of tumors from these mice found AAV integrations in *Rian*, which were not clonal, as well as clonal integrations in *Rtl1* (a gene in close proximity to *Rian*) and *Tax1bp1*.^49^ *Rtl1* and *Tax1bp1* are potential oncogenes, suggesting that integration into genes other than *Rian* could cause genotoxicity in mice, although the significance of these clonal rAAV integrations to tumor development was unclear because these integrations were found in OTC-deficient mice with a B6C3F1 hybrid background, which are known to have a high frequency of spontaneous liver tumors.

Another study that delivered rAAV to neonatal methylmalonic acidemia (MMA) and WT mice detected HCC in 50%–70% of mice by 2 years of age, in contrast to the background incidence of <10% in this mouse strain.^56^ AAV integration studies of tumor and normal liver tissue were performed. Clonal integration events were found in these tumors at the *Rian* locus, which caused an increase of transcription of genes proximal to these integrations, including micro-RNAs and *Rtl1*, recapitulating the original findings of Donsante.^46^ Common integrations not related to tumorigenesis, which included integrations in liver-specific genes *Alb* and *Afp*, were identified in this study. Of note, the *Alb* locus had more rAAV integrations than any other gene, including *Rian*. The high-dose group of mice in this study received a dose of ∼1 × 10^14^ vg/kg, which is similar to the dose approved for systemic rAAV delivery in humans to treat SMA type 1. This study found that rAAV genotoxicity is dose dependent and that some enhancer-promoters (CAG and thyroxin-binding globulin [TBG]) caused genotoxicity, while the human alpha-1 antitrypsin (hAAT) enhancer-promoter even when integrated into *Rian* did not cause tumorigenesis. The CAG and TBG elements were also used in other studies that observed genotoxicity.^42^^,^^48^ These findings suggest that vector design plays a role in toxicity and that vectors with reduced toxicity could be designed.

In a gene therapy study for Sandhoff disease, neonatal mice were given high doses (2 × 10^14^ vg/kg) of an rAAV9 vector expressing the hexosaminidase b-subunit under the control of the cytomegalovirus (CMV) promoter. Liver tumors developed in seven of 10 treated mice by 43 weeks with rAAV insertions in liver tumors that mapped to the *Rian* locus. A single treated mouse developed a lung tumor and was found to have an rAAV integration in fibroblast growth factor receptor 2 (*Fgfr2*), a gene known to show altered expression in some cancers.^57^ The pathology report from the liver tumors indicated benign hyperplasia, but did not rule out hepatocellular adenoma. The clonality of the rAAV integrations was not determined, so it is not clear if the rAAV integration played a role in tumor formation.

Recently, combination treatment for Krabbe disease involving an rAAV9-based gene delivery to the CNS was shown to cause almost 100% incidence of HCC in both WT mice and in the Twitcher (Twi) disease murine model.^60^ rAAV integrations in *Rian* were found in all the tumors analyzed. In addition, rAAV integrations were identified within or near genes that regulate cell growth, known or potential tumor suppressors, or associated with poor prognosis in human HCC. However, it should be noted that the ancillary treatments were likely to promote ds DNA breaks (DSB), and therefore increase rates of rAAV integration.^65^ The identification of potentially toxic integrations in addition to *Rian* integrations suggest that the *Rian* integration might represent one “hit” in a “multi-hit” process leading to tumorigenesis.^66^ However, targeted AAV-mediated delivery of the CAG enhancer-promoter element to the *Rian* locus resulted in a rapid progression of HCC formation in all mice after AAV treatment, suggesting that a second mutation may not be necessary for HCC formation.^53^

Together, these results document the risk of HCC in neonatal mice treated with AAV therapy and illuminate aspects of the mechanisms by which AAV integration caused HCC in mice (Table 1). Age of treatment, vector dose, and the vector enhancer-promoter structure also influenced the frequency of rAAV insertional mutagenesis. Although AAV integrations in *Rian* are commonly found to be the cause of insertional mutagenesis in mice, studies suggested that other genetic loci could also contribute to HCC as well as lung cancer. While not the primary cause of rAAV-mediated genotoxicity, some mouse studies suggest that auxiliary treatments and liver fibrosis could also increase the risk of AAV genotoxicity.

### The role of animal age

Studies in mice have shown that age plays a role in the occurrence of rAAV-mediated insertional mutagenesis. To date, the genotoxicity associated with rAAV integrations in the *Rian* locus is derived from studies that treated mice as neonates at day 1 or 2 postpartum.^42^^,^^46^^,^^56^^,^^57^^,^^60^ Increased rates of tumorigenesis, after treatment rAAV of juvenile/adult has been reported in WT mice treated with a *LacZ* vector but it is unclear if rAAV insertional mutagenesis was the cause of tumorigenesis in these mice.^48^^,^^49^ Conversely, several studies of large numbers of juvenile and adult mice treated with AAV failed to demonstrate AAV induced tumorigenesis, emphasizing the vulnerability of neonatal mice.^47^, ^51^, ^52^, ^55^, ^67^, ^68^

AAV integration events in the *Rian* locus have been associated with HCC in neonatal mice. *Rian* rAAV integrations might occur more frequently in mice because *Rian* expression is higher earlier in life^69^ and actively expressed genes are preferred locations of rAAV integrations.^17^^,^^40^^,^^69^ However, the other genes that are active early in life also represent common integration sites in mouse studies (*Afp*, *Alb*) but are found in both tumor and non-tumor tissue. In contrast, *Rian* is preferentially integrated in tumor tissue, suggesting a clonal selection within the HCC. Furthermore, rAAV integrations in the *Afp* locus, which is expressed at high levels early in life, have been reported to occur at higher frequencies than other genetic loci in mice treated with rAAV as neonates.^56^

The findings in mice suggest that tissues or animals treated with rAAV at times when genes involved in differentiation and cell cycling are actively expressed would be at greater risk of insertional mutagenesis. Unfortunately, the exact age at which mice might become less susceptible to rAAV genotoxicity is unclear as most of the studies that examine AAV genotoxicity were intended to evaluate the efficacy of gene delivery to treat genetic diseases and not assess rAAV integration-mediated toxicity. Another consideration is the developmental differences between a neonatal mouse and a human.^70^

### The effect of underlying liver disease

While liver disease did not play a role in the rAAV genotoxicity in neonatal mice described above, pre-existing liver disease may increase the risk of rAAV-related genotoxicity. Adult mice exposed to rAAV gene therapy in the context of chronic liver disease were also shown to develop HCC at high frequency,^59^ thus showcasing the need for additional studies in humans given the high prevalence of inflammatory liver disease in the population.

Induction of liver injury and cell proliferation in an adult mouse model that was administered an AAV vector that targeted the CAG promoter to the *Rian* locus by an AAV gene editing approach or a control reporter AAV resulted in development of HCC.^59^ In this study the mice were fed a high-fat diet, which induces liver injury, or underwent partial hepatectomy, which led to HCC in these adult mice. This demonstrates that hepatocyte proliferation and chronic liver injury can lead to rAAV-induced HCC in adult animals. These findings suggest that AAV administration to adult mice can lead to HCC in the context of chronic liver disease, a state that induces hepatocyte proliferation. This highlights that inflammatory liver conditions that include non-alcoholic fatty liver disease (NAFLD), non-alcoholic steatohepatitis (NASH), or hepatitis B and C infections may have a higher risk of developing HCC after AAV gene therapy.

### Limited studies of AAV-related tumorigenesis in rats

There are limited published studies in which rats have been administered rAAV gene therapy. A study evaluating AAV-induced oncogenesis in neonatal rats^71^ using a self-complementary (sc) AAV and strong ubiquitous CMV promoter did not find evidence of increased neoplastic potential or preferential sites of DNA integration. It was concluded that liver tumorigenesis after AAV treatment was absent in neonatal rats. However, the CMV promoter has limited activity in hepatocytes *in vivo*^72^^,^^73^ and the short 2-month observation period after dosing is insufficient for a thorough assessment of neoplastic outcomes.

### Evaluation of rAAV integration and HCC risk in non-human primates

Non-human primate (NHP) studies provide the opportunity to investigate the efficacy and safety of AAV gene therapy in a large animal model. To date, no genotoxicity has been reported in NHP studies, but almost all published studies consist of short-term observations, thus not allowing for long-term genotoxicity evaluation. Several studies have reported AAV integration analysis after AAV administration in NHPs (Table 2).^23^,^25^^,^^74^, ^75^, ^76^ Several studies have not detected a preference for gene coding regions and showed no clustering of integration sites,^21^^,^^25^^,^^77^ while others reported recurrent integrations around transcription units.^75^^,^^76^ The integration frequency is reported to be low, but there are methodological challenges in assessing this frequency.

**Table 2:** AAV integration studies in large animals

Publication	Species	Disease model	AAV vector; route	AAV dose (vg/kg)	Time of treatment	Duration of follow-up
Nowrouzi et al. 2012^23^	NHP	WT (n = 18)	AAV-RSV-LEA29Y AAV1 or AAV8 IV or IM	5 × 10^12^	adult	1.2–2.8 years
Gil-Farina et al. 2016^27^	NHP	WT (n = 6)	AAV5-hPBGD	1 × 10^13^ 5 × 10^13^	adult	1 month
Mattar et al. 2017^74^	NHP	WT (n = 6)	AAV-hFIX AAV8 or AAV5	1.4–1.9 × 10^13^	*in utero*	11–71 months
Spronck et al. 2020^75^	NHP	WT (n = 12)	AAV5-hFIX IV	5 × 10^11^ 5 × 10^12^ 2 × 10^13^ 9 × 10^13^	adult	6 months
Sullivan et al. 2021^76^	NHP	WT (n = 12)	AAV5-hFVIII IV	2 × 10^13^ 6 × 10^13^	adult	13 weeks and 26 weeks (6 months)
Niemeyer et al. 2009^78^	dog	hemophilia B (n = 4)	AAV2-CMV-cFIX (IM) AAV2-(Apoe)4/hAAT-cFIX (IV)	1.1 × 10^12^ to 3.4 × 10^12^	juvenile/adult 5.5–12 months	8 years
Nguyen, Everett et al. 2021^79^	dog	hemophilia A (n = 9)	AAV-TBG-cFVIII AAV-hAAT-cFVIII AAV8 or AAV9 PV or IV	1 × 10^13^ 2 × 10^13^ 4 × 10^13^	juvenile/adult 5 months to 4 years	2–10 years
Batty et al. 2020^80^ Batty et al., 2020^81^	dog	hemophilia A (n = 8)	AAV-TTR-cFVIII AAV2, 6, 8 PV	6 × 10^12^ to 2.7 × 10^13^	juvenile/adult 6 months to 2 years	8–12 years

IV, intravenous; IM, intramuscular; PV, portal vein.

One NHP study has provided the longest follow-up: almost 6 years (4–6 years) after a single intravenous injection of an sc AAV5 and AAV8 with an LP1-driven human factor IX (hFIX) transgene (scAAV-LP1-hFIXco) to late-gestation fetuses or adult animals.^77^^,^^82^ Sustained clinically relevant levels of hFIX with liver-specific expression were observed without any clinical concerns.^74^^,^^77^^,^^82^ Since AAV integration is more common in actively transcribed genes^17^^,^^40^^,^^52^ and there is very active transcription in early development,^83^ *in utero* AAV administration may result in more AAV integration events; however, currently there are no data to support this hypothesis. These data show promise for conditions where gene transfer can prevent perinatal death and long-term irreversible sequelae, but also highlight that lifelong surveillance is important.

### Long-term studies in dog models

AAV gene therapy studies are performed in dog models, which can provide the opportunity to evaluate efficacy and safety in a large animal model of a disease. Long-term follow-up studies in dogs have not reported AAV-related tumorigenesis. In a long-term study of AAV gene therapy in hemophilia A dogs,^84^ nine dogs were treated with AAV8 or AAV9 vectors expressing canine factor VIII (AAV-cFVIII) with the TBG or hAAT promoter either as a single chain or two chains and followed for up to 10 years.^79^ Unexpectedly, two dogs receiving AAV gene therapy began to have increased plasma FVIII levels starting 4 years after treatment.^79^^,^^85^ AAV integration analysis on the liver tissue of these two dogs and four others revealed that vector integration in the host genome correlated with vector copy number, and clonal expansion of some cells harboring integrated AAV genomes was detected. The basis of the increase in FVIII levels is unknown, but one speculative model proposes that cell clones expanded harboring integrated genomes with intact FVIII coding regions, and these delivered slowly increasing levels of FVIII to the circulation in two dogs. None of the dogs in this study showed evidence of tumors or altered liver function revealed by liver enzyme levels and serum alpha-fetoprotein (AFP), a clinical biomarker for HCC. Results of a second long-term study in hemophilia A dogs treated with AAV-cFVIII delivered in AAV2, AAV6, and AAV8 with the liver-specific transthyretin (TTR) promoter were presented at the roundtable, and in this case also, no transformation was detected in dog liver. Integration site analysis was reported to be underway and additional data should soon be forthcoming.^80^^,^^81^

In hemophilia B dogs, long-term follow-up after treatment with a variety of AAV serotypes^78^, ^86^, ^87^ did not show signs of neoplasia. These studies included 135 hemophilic dogs, injected (intravascular, intramuscular, or isolated limb perfusion) with AAV vectors and followed for longer than 10 years. No evidence of AAV-associated tumor formation has been found in liver or skeletal muscle as judged by ultrasound and/or necropsy. However, no AAV integration studies were performed on these dogs, so it is unknown if there was any clonal expansion. Most of the dogs were injected as juveniles (1.5–10 months of age) with the vectors containing the cytomegalovirus (CMV) promoter for expression in muscle or hAAT promoter for liver-targeted expression suggesting that the combination of these promoters and liver growth does not result in a high-level neoplastic risk in dogs.

AAV gene therapy with vectors encoding glucose 6-phosphatase (G6Pase), which is an enzyme that hydrolyzes glucose 6-phosphate, reversed underlying hepatocellular abnormalities in dogs with glycogen storage disease (GSD) type Ia.^58^ These dogs, similar to humans with GP6ase deficiency, are high risk for developing hepatocellular adenoma (HCA). Some dogs developed HCC after treatment and G6Pase activity was found to be lower in the tumor tissue in comparison with adjacent liver. Additional tumor marker analysis revealed that the dog tumors did not lead to elevated expression of alpha-fetoprotein, which is a marker associated with HCC in mice that occurs after AAV vector integration.^24^^,^^56^ Therefore, AAV-G6Pase vector was concluded not likely to be the cause of HCC in the GSD Ia dogs. Instead, it is assumed that AAV administration prevented HCC development by correcting the underlying disease in these dogs.^58^ However, AAV integration analysis of the tumors and neighboring tissue was not performed. In the same report, GSD Ia mice were also treated and AAV integration and tumor marker analysis in mice revealed that HCC arose from GSD Ia, and not from vector administration.

## Global sites of AAV vector genome integration

In most studies, the integration sites of rAAV are typically found near active transcription units and associated annotation. It has been observed that integration events are more prevalent in CpG islands and GC-rich regions, which are associated with active transcription.^17^^,^^56^^,^^52^^,^^79^^,^^88^ Integration events are recovered more commonly in some regions. Enrichment can be due to either favored initial integration or favored cell proliferation and survival. Some data suggest that there are preferences for integration in specific sites. Neonatal mouse studies suggest that not only *Rian* but also albumin (*Alb*) and alpha-fetoprotein (*Afp*) were more frequent targets for AAV integration. Of 1,205 loci with rAAV integrations, 149 had multiple integration events.^25^ Canine studies also found some loci that had multiple integration events in multiple dogs, including *EGR2*, *EGR3*, *CCND1*, *DUSP1*, and *ALB*, with three of these sites (*EGR2*, *EGR3*, and *CCND1*) associated with expanded clones, suggesting that integration in these loci resulted in a cell proliferation or persistence.^79^ Studies in primates and human hepatocytes have also reported multiple rAAV integrations in *ALB*, suggesting that some rAAV integration sites are common across species.^27^^,^^59^ Additional data from different species will be required to determine if there are integration site preferences and whether data in animal studies are predictive of any observations in humans.

## Assessing clinical risk of insertional mutagenesis

HCC associated with AAV therapies has not been detected in humans to date; therefore, the risk factors for tumorigenesis, if any, are unknown. A long-term follow-up of hemophilia B patients for 6 to 15 years post administration of AAV2-hFIX, which included patients with co-morbidities that would predispose them for HCC development, reported no evidence of sustained hepatic toxicity or development of HCC as assessed by liver transaminase values, serum α-fetoprotein, and liver ultrasound.^89^ These are the longest available longitudinal follow-up data of subjects who received intravascular AAV delivery. Another hemophilia B clinical study that delivered scAAV8-LP1-hFIXco reported follow-up of 8 years in 10 subjects without clinical concerns.^90^ Ongoing hemophilia clinical studies also support the preliminary safety of intravascular AAV administration at the doses tested in adults (2 × 10^11^ to 6 × 10^13^vg/kg).^89^, ^90^, ^91^, ^92^, ^93^ Several different AAV serotype vectors (AAV1, AAV2, AAV5, AAV8, and AAV9) have or are currently being used in clinical trials (including in children) and no increase of cancer of any type has been reported.^34^^,^^94^

The studies of AAV integration after human clinical gene therapy studies are very limited due to the need to use invasive procedures in order to obtain tissue biopsies. Analysis of human muscle biopsies after the delivery of an rAAV1 encoding for the LPLS447X gene revealed that vector genomes were heterogeneously distributed throughout the tissue with detectable levels of vector integration.^95^ Integration sites were distributed across the host genome with main integration hotspots within mitochondrial genes and nuclear mitochondrial DNA regions. In a study investigating AAV persistence in the human liver, liver biopsies from acute intermittent porphyria patients receiving an AAV5 vector were analyzed.^27^^,^^96^ The study confirmed that AAV integration was both low in frequency and distributed genome-wide, with no clustered integration sites near genes that had been previously implicated in the mouse studies. Nonetheless, the number of integration sites retrieved in those samples was small, most likely because patients received a sub-therapeutic dose and little material was used as input, thus hampering the ability to draw conclusions and establish a comparison with preclinical datasets.

In December 2020, the FDA placed uniQure’s AMT-061 (etranacogene dezaparvovec), under investigation to treat hemophilia B, on clinical hold following the submission of a safety report of a possibly related serious adverse event associated with a preliminary diagnosis of HCC in one patient in the HOPE-B trial.^97^, ^98^, ^99^ The HCC was detected 1 year after dosing during a routine abdominal ultrasound that was part of the follow-up assessment for the study. It should be noted that the patient had multiple risk factors associated with HCC, including a 25-year history of hepatitis C virus (HCV) and hepatitis B virus (HBV) history, evidence of NAFLD, smoking history, familial cancer history, and advanced age.^98^^,^^100^ Consequently, in April 2021, the FDA lifted the study hold following multiple analyses conducted by an independent laboratory and review by leading external experts in the field, which showed that AMT-061 was unlikely to have contributed to the HCC in the patient.^98^^,^^101^ Notably, the press release highlighted that AAV vector integration in the patient’s tissue sample was extremely rare,^101^ although there are challenges in accurately estimating the number of vectors integrated in gene-modified cells using DNA sequence information.^102^ The integration events were reportedly distributed across the genome with no evidence of clonal expansion or any dominant integration event. However, the similarities between the development of HCC in a mouse model of chronic liver injury after AAV administration^59^ and the co-morbidities in the hemophilia B patient should not be ignored. The study results reported in the uniQure press release are yet to be publicly accessible, which further supports the need for data transparency and sharing in AAV research and in the rapidly growing gene therapy space at large.

### Technologies and challenges of assessment of AAV integration

Integration events generated after AAV gene therapy are commonly quantified using next-generation sequencing (NGS).^27^^,^^103^, ^104^, ^105^ At different stages post dosing, the genomic DNA is isolated from animal tissues and insertion events in the host cell genome can be recovered using various technologies. Techniques such as ligation-mediated PCR (LM-PCR) or linear amplification mediated PCR (LAM-PCR), in combination with high-throughput sequencing, are able to detect from hundreds to thousands of integration sites from a single sample.^27^^,^^103^, ^104^, ^105^ Novel PCR methods for integration site retrieval that adopt physical DNA fragmentation methods to avoid the amplification biases produced by the uneven fragmentation of genomic DNA with restriction enzymes allow the quantitative retrieval of vector integration sites for accurate clonal quantification estimates.^106^^,^^107^ The complexity of AAV rearrangement at the site of integration can complicate analysis.^79^^,^^88^ Non-PCR-based methods for the quantitative integration site retrieval, such as those based on the capture and enrichment of viral/host genome DNA junctions, provide excellent results, although sensitivity might not be comparable with that achieved by PCR-based methods.^95^ Nonetheless, systematic comparative studies are still missing. The main advantage of target enrichment-based approaches^108^ is that they do not require the presence of a primer binding site, thus enabling the retrieval of truncated or internal vector regions that get integrated into the host genome. In addition, this target enrichment-based approach also allows capture of large DNA fragments, which can be subjected to long-read sequencing to study rearrangements of the AAV genome in preparations and in tissue samples.^88^^,^^109^^,^^110^ After the integration sites are identified in the tumor-infiltrated tissue, the host genes in the proximity of insertion sites may be analyzed for expression changes that may lead to biological dysfunctions and cancer risk.^111^ However, unless there is a clear clonal dominance of the clone of interest within the analyzed cell pool, gene expression analyses can be challenging due to masking by the heterogeneity of the cells contained in the tumor.

Where studied, integrated rAAV DNA is often found to be rearranged, and this is also true of rAAV DNA in vector particles prior to transduction.^1^^,^^109^^,^^110^ Hence, there is a need for sequence-based characterization of vector genomes present in AAV batches as well as those persisting within the tissues after transduction.^111^ Technologies for elucidating any rearrangements in vector preparations are nascent.^109^^,^^110^^,^^112^ The use of NGS to characterize rAAV preparations may be challenging, in part because the vector DNA is single stranded, whereas in tissues the DNA has been converted to dsDNA. Heavily rearranged AAV genomes and even plasmid sequences have been found to be integrated in the genome of human hepatocytes transduced *ex vivo* or *in vivo*.^88^ The molecular characterization of AAV vector preparations will be important for understanding the potential impact that this could have on genotoxicity risk.

It is of interest to estimate the numbers of cells harboring integrated vectors after rAAV gene transfer, but all methods are subject to uncertainties. It is, of course, possible to count the number of different integration sites in a sequence sample, but this rarely captures the whole population. More typically, integration site analysis provides a sample of the population, and not the whole population. Thus, mathematical methods are needed to estimate the population size from partial samples.^102^^,^^107^^,^^113^ There are examples of estimates in large animal models and humans.^27^^,^^76^^,^^81^^,^^88^^,^^102^ Other approaches are also possible. For example, for cells that have proliferated extensively after gene addition, unintegrated forms will be mostly diluted out, so that simple qPCR for vector sequences provides a measure of the total numbers. However, this only quantifies the number of sequences complementary to the PCR amplicon, and so the proportions of intact versus rearranged vectors is not reported by this measure. This is an important point, given that high proportions of AAV vectors associated with integration are found to be rearranged.

Development of novel methods for detecting AAV integration events and clonal expansions may provide tools for assessing risk in human subjects. Cell-free DNA (cfDNA) that is isolated from peripheral blood has also been identified as a potential safety biomarker that allows for early identification of expanding clones growing in solid organs that are released into the bloodstream from apoptotic or necrotic cells.^114^^,^^115^ This approach applies an LM-PCR approach with high-throughput sequencing similar to that used for solid tissues. One unique aspect of this analysis is that it may provide a tool for following a subject over time using non-invasive procedures. Preliminary results show that AAV integrations can be retrieved from cfDNA, but further research and validation is needed.^1^^,^^4^^,^^114^^,^^115^

Novel models that use human hepatocytes may provide additional insights into AAV integration profiles. A humanized liver mouse model provides a tool to analyze *ex vivo* or *in vivo* AAV transduction of human hepatocytes.^1^^,^^88^ One limitation of this model is that it involves extensive proliferation of hepatocytes to repopulate the liver, which may influence AAV integration frequency. Recent studies in this model using a targeted capture approach and long-read sequencing resulted in a limited number of sequences but showed extensive rearrangements of the AAV vector genome and integration profiles that demonstrate that this model may be valuable in characterizing AAV integration events.

Nuclease-based genome editing platforms delivered using rAAV vectors as well as AAV vectors specifically designed for nuclease-free genome editing are being developed for targeted correction of disease-causing mutations.^116^ While the goal of this approach is targeted genome editing, the frequency of random AAV integration in this context is not known and will require additional analysis.

## Considerations based on experience with retroviral-based gene therapies

More than 20 years of clinical studies and long-term follow-ups with AAV have not demonstrated any toxicity related to integration,^2^^,^^34^^,^^89^, ^90^, ^91^, ^92^, ^93^ but genotoxicity associated with retroviral (RV) vectors is well characterized.^117^, ^118^, ^119^, ^120^, ^121^, ^122^, ^123^, ^124^ Recent data on retroviral vectors in humans support four genetic mechanisms of insertional mutagenesis:^119^ (1) gene activation by integration of an enhancer sequence encoded in a retroviral vector (enhancer insertion), (2) gene activation by promoter insertion, (3) gene inactivation by insertional disruption, and (4) gene activation by mRNA 3′ end substitution. In each scenario, integration in patients might be associated with clonal expansion or genuine transformation depending on the affected gene(s).

The challenges of assessing the clinical risk of oncogenesis are illustrated by the early gamma (γ)-RV-based hematopoietic stem cell gene therapy trials for X-linked severe combined immunodeficiency (SCID-X1), Wiskott-Aldrich syndrome (WAS), chronic granulomatous disease (CGD), and adenosine deaminase (ADA-SCID). In these trials, some patients developed leukemia originating from transplanted cells in which cellular oncogenes were activated by the strong enhancers present in the long terminal repeats (LTRs) of γ-RV vectors integrated nearby.^118^,^120^,^122^^,^^124^, ^125^, ^126^, ^127^, ^128^, ^129^ These adverse events were unexpected as preclinical testing in WT mice and disease mouse models did not show any evidence of oncogenic transformation.^129^, ^130^, ^131^, ^132^, ^133^, ^134^ The failure to detect the genotoxic potential of γ-RVs may be explained by the difficulty of inducing cell transformation in these models.^135^, ^136^, ^137^, ^138^ Because full malignant transformation is achieved only after the acquisition of multiple mutations, the lifespan of mice could be too short to allow full transformation to occur. Also, the number of cells that are typically transduced in mouse models of gene therapy are significantly less than the number of cells modified in human patients, thus reducing the probability to detect cancer-triggering insertions.

These unexpected adverse events have prompted the development of novel preclinical assays such as *in vitro* cell immortalization assays and *in vivo* oncogenicity studies based on highly sensitive tumor-prone mouse models to test the genotoxic potential of different RV vector types and designs.^63^^,^^123^^,^^139^, ^140^, ^141^, ^142^, ^143^, ^144^, ^145^ The advantage of using tumor-prone mice, such as the Cdkn2a-deficient mice, is that they are highly sensitized to insertional mutagenesis and easily develop tumors, while in WT mice the tumor onset is only sporadic at best. Indeed, by transplanting vector-transduced Cdkn2a-deficient hematopoietic stem and progenitor cells into WT mice, it was possible to measure and compare the genotoxic potential of γ-RVs and lentiviral vectors (LVs) with self-inactivating (SIN) LTRs.^142^ The positive safety data obtained with SIN LV LTRs have greatly reduced the concerns related to insertional mutagenesis and guided the choice of vector platforms.^145^, ^146^, ^147^, ^148^, ^149^, ^150^, ^151^

Mouse models were also developed to test the genotoxicity of LV vectors used for liver-directed gene therapy, allowing the field to understand what vector features have the most profound impact on safety.^63^^,^^123^^,^^152^ These studies indicated that the position and strength of the promoter within the vector backbone had a strong effect on genotoxicity, while the integration profile was less of a determinant. These models also documented that the inclusion of chromatin insulator sequences within the LV LTRs reduces the risk of enhancer-mediated oncogene activation and oncogenesis *in vivo*. Thus, although mice may have limited translatability of risk to humans, they are informative for the choice of the best-performing vector design.

## Evaluating the relationship between HCC risk and vector design

The risk of HCC in animal models has been suggested to correlate with AAV vector dose and the nature of the enhancer and promoter elements of the transgene. Data have demonstrated that the integration of promoter and enhancer sequences alone can drive tumorigenesis.^53^ In a study conducted in HCC-prone C3H/HeJ mice,^54^ partial hepatectomy after AAV infection, which is supposed to induce cell cycling, reduced HCC incidence in the CMV-GFP-infected mice, but not in the chicken beta-actin (CBA)-null-infected group. Tumors from CBA-null-infected, hepatectomized mice were more likely to contain significant levels of vector DNA than tumors from the corresponding CMV-GFP-infected group, implicating the stronger tumorigenesis capability by CBA promoter than by the CMV promoter. However, the CMV promoter is not very active in the liver.^72^^,^^73^^,^^153^ Most CBA-null vector insertions recovered from tumors were associated with known proto-oncogenes or tumor suppressors, suggesting strong readthrough transcription, enhancer effects, and disruption of tumor suppressors by stronger promoters.

Experiments in mice suggest that the use of certain liver-specific promoters versus ubiquitous promoters reduces the risk of tumor formation as a consequence of integration.^56^ In the GSD Ia mice administered AAV vector,^58^ *Rian* AAV integrations but not the upregulation of gene expression in *Rian* loci were detected. It was hypothesized that the tissue-specific promoters included in the vectors, the liver-specific promoter LP1 and minimal G6PC promoters, failed to activate the *Rian* locus, as has been described for a liver-specific hAAT promoter.^24^^,^^25^^,^^56^^,^^58^ Thus, it is believed that the strong promoters, such CAG or TBG promoters, likely drive the readthrough after genome integration and trigger the nearby proto-oncogene expression that leads to HCC development.^53^ Furthermore, studies have also shown the presence of a 46-nucleotide liver-specific enhancer-promoter element within the WT AAV2 genome in the region located between the *cap* gene and the right ITR.^154^ This sequence is also conserved in some of the AAV vectors and, therefore, could contribute to dysregulation of expression upon vector genome integration. The hAAT vector that did not cause upregulation of proximal genes or toxicity contained the WT AAV enhancer element, suggesting that the WT AAV enhancer element alone is not sufficient to cause tumorigenesis.^56^

An analysis of canine specimens following AAV administration with liver targeting TBG or hAAT promoters reported integration events with evidence of clonal expansion without tumorigenesis.^79^ This study used constructs that contain the 46-nucleotide enhancer element that resides in the AAV2 ITR,^154^ but it is not known if this influenced clonal expansion observed in this study. Furthermore, hemophilia B dogs treated at young age with a variety of AAV vectors containing the CMV or hAAT promoters did not show signs of neoplasia after 10 years of follow-up.^78^, ^86^, ^87^

In addition, the human translation of promoter effects on HCC risk after AAV administration is still unknown.^3^^,^^59^ However, an improved understanding of promoter effects has led to redesigned retroviral vectors that have an improved safety profile. As such, it will be important to ascertain potential promoter effects or relative risks of promoter elements using sensitive animal models of oncogenesis despite any limitations in its translatability to humans.^4^^,^^56^ Although further studies on the oncogenesis of strongly transactivating vectors in large animals will be highly informative, it is important to point out that these studies are quite impractical as an imminent solution for future AAV gene therapy development. Moreover, it may take years of research to build and develop robust and rigorous data.

## Evaluating the correlation between dosing and HCC risk

Data from mice suggest that AAV vector doses may be a contributing factor to HCC risk; however, the translation of findings to humans is unclear. A study in neonatal mice showed a significant decrease in the rate of tumorigenesis as the rAAV dose of the same vector was decreased.^56^ Typically, rAAV gene transfer in neonatal mice uses high vector doses in the order of 1 × 10^14^ vg/kg or higher to counteract the dilution of episomal AAV DNA from hepatocyte proliferation.^42^^,^^44^^,^^46^^,^^56^ There is a correlation between AAV vector dose and vector copy numbers in target tissues, and higher vector copy numbers are associated with more integration events.^79^^,^^88^

In contrast to findings in mice, high AAV vector doses have been investigated in clinical trials; however, to date, no cases of HCC have been reported. Doses spanning the range of 1 × 10^14^ to 3 × 10^14^ vg/kg have been used in recent clinical trials (e.g., NCT03199469, X-linked myotubular myopathy), as well as for the approved Zolgensma (onasemnogene abeparvovec-xioi), both targeting a very young population (infant to toddler). Other gene therapy clinical trials are using doses in the range of 1 × 10^12^ to 6 × 10^13^ vg/kg (NTC02991144, NTC04088734, NCT03001830, NCT02576795, NCT01620801). An ongoing, 15-year, observational, long-term, follow-up Zolgensma study enrolled a total of 13 infant patients, 10 from the high-dose (therapeutic) cohort and three from the low-dose cohort. It was recently reported that a favorable safety profile was observed for up to 6.2 years after dosing.^33^ To date, over 1,800 patients have been treated with Zolgensma, representing a large cohort of AAV-treated subjects to follow.^155^ Long-term data across clinical trials investigating therapeutic AAV delivery will be important to support the understanding of the impact and the risk in humans.

## Broad population-based risk assessments

Understanding the role of the underlying disease and other risk factors that can affect genotoxicity is essential. Major risk factors for HCC include chronic alcohol consumption, diabetes or obesity-related non-alcoholic steatohepatitis, and infection by HBV or HCV.^156^ NAFLD and previous infections with HBV and HCV that are common in certain populations, such as hemophilia, are also associated with a higher risk for HCC.^157^ Chronic infections with HBV and HCV have been associated with approximately 80% of HCC cases.^157^ The incidence of HCC in a subject in the HOPE-B clinical trial for AAV-FIX delivery for hemophilia B, as described in detail above (section “assessing clinical risk of insertional mutagenesis”), illustrates the challenges of assessing the cause of the HCC in a subject with other risk factors.^100^^,^^102^ Again, highlighting the importance of long-term monitoring and methodical investigation into the cause of the HCC.

## Cancer and natural AAV infection

Of note, natural AAV infection, mainly AAV2, is ubiquitous in humans, with roughly 80% of adults harboring neutralizing antibodies directed against AAV2.^158^ However, no specific diseases have yet been associated with natural infection. Additionally, the magnitude of WT AAV’s genotoxic potential is also unclear considering the prevalence of natural infections and the low percentage of HCC cases bearing an eventual AAV-related event.^159^ While studies to investigate WT AAV infections and HCC have detected AAV DNA integrated in HCC tumors as well as in normal liver tissue, the role of these integration events in tumorigenesis is not well understood given that, in many cases, the AAV integration event was not present in all the cells in the tumor.^159^^,^^160^ Thus, the relevance of WT AAV integration with rAAV gene therapy vectors is unclear and has been challenged.^161^, ^162^, ^163^ Hence, the widespread seroprevalence in humans of WT AAVs without clear association with any disease, including neoplasia, supports the conclusion that rAAV therapeutic vectors have minimal potential to induce tumors. However, there is currently limited understanding of natural AAV infections in humans, and no data on the circulating virus load or how this would compare with a gene therapy dose of rAAV.

## Summary of expert recommendations

In addition to the expert roundtable convened in August 2021, ASGCT also conducted three group interviews with eight leading experts to generate insights from clinicians and researchers on AAV integration, including oncogenesis in patients, clonal expansion, bioethics, technical challenges of studying AAV integration, and projections and considerations for the future. These experts, along with the authors on this publication, underscore the importance of alignment on definitions such as the copy number and number of integration sites. Additionally, there is also a need to better understand how the data today align with expansions that were previously observed in simple clonal models while also accurately measuring the number of integrants regardless of analytical method. A summary of additional expert concerns and recommendations are summarized in Table 3.

**Table 3:** Summary of expert concerns and recommendations

Expert concerns/observations	Expert recommendations
There are insufficient data to fully delineate the magnitude of cancer risk; hence, efforts should be made to collectively aggregate, share, and analyze data	• increased focus on the development of standard assays using sequencing based technologies, with acceptable quality-control metrics on reproducibility, sensitivity, and specificity • build and develop unified standards with aggregate data to assess translatability, including consistent use of positive and negative controls • need for alignment on the definition of a clonal expansion • encourage the use of transparent open-source software and universal data sharing • data on potential integration-related severe adverse events should be aggregated in publicly available archives
There is concern surrounding the use of animal models to model potential genotoxicity and whether the findings would be translatable to humans	• animal data need to be well recorded and analyzed for similarities and differences in humans • risk quantification should also delineate the vector type and design
Routine liver biopsies without medical indication should be avoided	• follow-up should include ultrasound scans or MRI and AFP • focus should be on analysis of any tumors detected after gene therapy. Notably, the amount of tissue in a biopsy may not be representative of the whole organ, and would likely be uninformative, containing only randomly integrated AAV, with the exception of tumor biopsies
Given the current lack of observed genotoxicity in large animal models and humans, should patients still be informed and are physicians properly aware of what and when to communicate?	• while AAV-related tumorigenicity has been observed only in mice, vector integration has been observed and measured in large animals and humans, and rAAV must therefore be considered a mutagen • there is a distinct need for bidirectional patient and physician education on the need for informed consent

This summary is based on interviews with the experts Drs. Jessica Zucman-Rossi,^1,2,3^ Graham Foster,^4^ Frederic D. Bushman,^5^ Phillip W.L. Tai,^6^ Richard Gabriel,^7^ Mark A. Kay,^8^ Eugenio Montini,^9^ and Charles Venditti.^10^

^1^Centre de Recherche des Cordeliers, Sorbonne Universités, INSERM, Paris, Île-de-France, France.

^2^Functional Genomics of Solid Tumor, Labex Immuno-Oncology, équipe labellisée Ligue Contre le Cancer, Université de Paris, Université Paris 13, Paris, Île-de-France, France.

^3^Department of Oncology, APHP, Hospital Européen Georges Pompidou, Paris, Île-de-France, France.

^4^Centre for Biomedical Sciences, School of Biological Sciences, Royal Holloway University of London, Egham, Surrey, UK.

^5^Department of Microbiology, Perelman School of Medicine, University of Pennsylvania, Philadelphia, PA, USA.

^6^Horae Gene Therapy Center, Li Weibo Institute for Rare Diseases Research, Department of Microbiology and Physiological Systems, University of Massachusetts Medical School, Worcester, MA, USA.

^7^ProtaGene CGT GmbH, Heidelberg, Germany.

^8^Departments of Pediatrics and Genetics, Stanford University, Stanford, CA, USA.

^9^San Raffaele Telethon Institute for Gene Therapy, IRCCS Ospedale San Raffaele Scientific Institute, Milan, Italy.

^10^National Human Genome Research Institute, National Institutes of Health, Bethesda, MD, USA.

### Relevance of animal models

Evidence for AAV genotoxicity and carcinogenicity have been generated primarily from neonatal mice or mice with conditions prone to cancer development. The risk of rAAV-associated liver tumorigenesis does not seem to translate into older mice in the absence of pre-existing liver disease or large animal models such as dogs and NHPs. Although AAV integration and clonal expansion have been observed in hemophilia dogs after long-term follow-up after AAV treatment, it is currently unknown if the clonal expansions detected in the dogs were pre-malignant and could result in malignancies if the dogs had lived longer. The canine studies highlight the importance of long-term follow-up. However, no animal model is fully translatable and there are no other species that can be reasonably studied as long as humans.

Despite their extensive use in biomedical research and drug development, it is clear that animal models can be poor predictors of human disease. rAAV treatment-related integration and tumorigenesis risk in humans needs further evidence from patient populations. Integration profiles for both humans and animals show wide distributions on chromosomes and commonly modest positive association with transcription. However, only a few human subjects have been evaluated for rAAV integration analysis, thus continued collection of integration data is needed, as is consistent with patient welfare. Studies of integration profiles in human samples will typically only represent a snapshot in time and localization of the tissue. Furthermore, clinicians would need to ascertain what to do with the analyzed data and the potential impact, if any, on clinical decisions. Taken together, AAV gene therapy safety in patients should be closely monitored in clinical development and post-marketing patient care for any signs of tumor development.

### A database of cancer cases in gene therapy recipients

The authors of this review also suggest that improved databasing of cancer cases in gene therapy subjects would be useful. Thousands of people have now been treated with gene therapy. Aging humans commonly develop cancer, so we must expect a steady rate of cancer cases in gene therapy recipients, but whether gene therapy contributed to their cancers is an unknown and an outstanding question. Already the field has worked through multiple cases and concluded that, for some cases of retroviral gene therapy, the treatment indeed contributed to cancer, whereas for rAAV there have been no confirmed cases of cancer. A detailed compilation of information on each such case, including co-morbidities, the data evaluated, and the decision reached, would greatly aid in determining an approach to assess causality in future cases.

## Regulatory guidance

The risks of AAV vector genotoxicity and carcinogenicity from insertional mutagenesis remain an ongoing concern for health authorities even though FDA and EMA guidance documents consider AAV vectors are non-integrating.^164^ For example, the FDA’s long-term follow-up guidance, finalized in January 2020, states that AAV vectors do not have the propensity to integrate, thereby presenting a lower risk of adverse events.^165^ However, both agencies discuss research publications by Donsante in 2001 and 2007 that described the induction of HCC, associated with AAV vector DNA integration, in mice that were treated with AAV as neonates and imply that this should be considered in safety evaluations.^42^^,^^46^

Historically, regulators have acknowledged that AAV integration is low risk based on publicly available review documents from the EMA and FDA on Glybera, Luxturna, and Zolgensma.^28^, ^29^, ^30^, ^31^, ^32^ For example, no direct integration testing was required to support the Luxturna approval; however, according to review documents, its manufacturer, Spark Therapeutics, justified not conducting genotoxicity studies partly on the basis that the current scientific literature reported a low integration frequency of AAV vectors and integration profile of AAV vectors.^166^ In addition, Spark justified not conducting development and reproductive toxicity (DART) studies based on the biological attributes of AAV2-hRPE65v2, its pharmacology and toxicology data, and the current scientific publications.^23^^,^^95^^,^^167^

For AAV vectors, the FDA recommends implementing a long-term follow-up protocol of up to 5 years leveraging a risk-based approach. This recommendation is in contrast to vectors known to be integrating (e.g., LVs), where the FDA recommends a long-term follow-up duration of 15 years. Nonetheless, manufacturers of approved AAV delivery products have incorporated a more conservative approach in recent studies. For example, Luxturna and Zolgensma, both approved, have incorporated a planned long-term follow-up period of 15 years.^166^ These post-marketing data, and additional safety and long-term follow-up data generated from ongoing clinical trials and long-term preclinical studies for various AAV vectors, will add to the body of knowledge to further define the safety profile for AAV vector gene therapies based on clinical data.

Recent preclinical^168^ and clinical studies^97^^,^^98^^,^^101^ that observed HCC after AAV administration provide insights into the regulatory perspective on AAV integration. After a subject in a hemophilia B clinical study presented with HCC, the trial was placed on hold while additional studies were performed to determine if AAV was involved in the HCC event, which determined that the AAV was not likely the cause of the HCC in this patient.^97^^,^^98^^,^^101^ More recently, adenomas and an HCC were observed in an interim preclinical study to assess durability after AAV5-PAH in phenylalanine hydroxylase (PAH)-deficient mice, resulting in a clinical hold on the phase 1/2 study.^168^ In both cases, the FDA requested additional investigation into these studies.

The topic of AAV integration continues to be one of importance to researchers, regulators, and patients alike. This is shown by the plethora of ongoing regulatory discussions on the topic, such as the September 2021 FDA CTGTAC meeting,^2^ the ASGCT Policy Summit,^3^ and the November 2021 ASGCT-FDA Liaison Meeting.^4^ Of note, several of the experts included in this publication contributed to the August virtual ASGCT roundtable meeting,^1^ as well as the FDA CTGTAC meeting.^2^ In addition, the 2021 ASGCT Policy Summit^3^ offered a variety of stakeholder perspectives on relevant topics in the gene therapy field and how they inform regulatory, legislative, and payment policies for diagnosis and treatment. During the Summit, Dr. Doug McCarty presented a summary of the ASGCT AAV Integration Roundtable.

While health authority guidance and recent meetings have been enlightening, additional clarity, perhaps incorporating some of the expert recommendations, would be useful (see Table 3 and Table 4). Recommendations are needed regarding interpretation of findings related to clinical development, overall impact on benefit-risk assessments for products, characterizing the theoretical risk to increase patient awareness, and also approaches to monitoring both in the clinic as well as with broader post-approval use.

**Table 4:** Summary of recommendations from the 2021 ASGCT-FDA Liaison Meeting

Criterion	Expert recommendations
Theoretical risk	• the risk of oncogenesis associated with rAAV chromosomal integration in humans is theoretical based on current data and information • the current lack of observed rAAV-associated HCC in large animals and humans suggests that the risk is low compared with neonatal mice and does not outweigh the potential benefit from many AAV vector-based gene therapies, especially for serious and life-threatening conditions with no alternative therapy • given the current diseases targeted with AAV gene therapy, and the actual documented risks, the risk/benefit ratio would suggest that many trials should be able to proceed in parallel with additional investigation
Animal models	• the use of sensitive mouse models of oncogenesis may provide information about the relative risk of different vector designs; however, translatability of any murine oncogenic events to humans remains unclear • if longer-term pharmacodynamic studies are conducted, the background rates of HCC must be considered, and risks must be understood • while additional studies assessing the oncogenesis of strongly transactivating vectors in large animals will be needed to better understand these issues, these studies are impractical as a near-term solution and may require one to two decades of research • recent observations of clonal expansions of rAAV integrations in canine hepatocytes in the absence of tumor formation warrant additional studies in the field. These may or may not reflect natural clonal dynamics in aging animals, and additional studies could help determine the nature of these clonal expansions
Methods for assessment	• performing integration site analysis, whole-genome sequencing, and gene expression analysis on tumor tissue samples from patients • limitations to these methods may potentially be mitigated by rapid and ongoing improvements in sequencing technologies such as target enrichment and long-read sequencing methods. These methods may help provide greater insight into concatemeric and highly rearranged integrated vector genome structures • propose that an expert group should be convened to make recommendations regarding improving methods used to assess oncogenic events • cfDNA should be further evaluated as a potential safety biomarker
Clinical assessment	• biopsies of neoplastic nodules and surrounding non-tumor tissue should be conducted to further investigate any positive signal picked up by non-invasive methods • long-term follow-up studies should last for at least 5 years, consistent with FDA guidance, and possibly longer depending on the safety profile of the product. Enrollment in patient registries for longer-term data collection is also encouraged

## Conclusions

The success of AAV gene transfer is best demonstrated by the recently approved gene therapy products (Luxturna, Zolgensma, Glybera). Additionally, the abundance of preclinical and clinical research on rAAV-based vectors delineates the need to ascertain its durability and clinical safety. Overall, the frequency of AAV integration is low and the risk of malignancy appears to be theoretical given that no cases of cancer associated with rAAV have been reported in humans to date.

While these discussions around AAV integration have focused on the implications of systemic and liver-targeted AAV delivery, there is less known about AAV integration in other tissues, such as the brain, muscle, or eye. The mitotic potential of any cell as well as other factors, including dose and vector design, may influence AAV integration and the potential for insertional mutagenesis. Thus, the investigation of the safety and potential genotoxicity of AAV delivery in other tissues will also be important to further define the safety profile of AAV gene therapy.

The ASGCT AAV Integration Roundtable as well as other recent meetings have brought the discussion of AAV integration and the need to investigate and better understand these findings to the forefront. While a recent case of HCC in a subject in a hemophilia B clinical trial did not find an association between the HCC and AAV integration, it raises the importance of this ongoing discussion on AAV integration and the risk of genotoxicity. The field must be committed to increased data transparency and sharing, investigation of the underlying mechanisms, and developing new tools and models for improving our understanding of the biological consequences of AAV gene therapy with the goal of minimizing any risk and ensuring that patients can benefit long term from these novel therapeutics.

## References

[1] ASGCT (2021). AAV integration roundtable. https://asgct.org/events/aav-roundtable?_zs=oAJHb&_zl=TNwf2.

[2] FDA (2021). https://www.fda.gov/advisory-committees/advisory-committee-calendar/cellular-tissue-and-gene-therapies-advisory-committee-september-2-3-2021-meeting-announcement.

[3] ASGCT (2021). https://asgct.org/advocacy/policy-summit/2021-agenda.

[4] ASGCT-FDA (2021).

[5] Lundstrom K. (2018). Viral vectors in gene therapy. Diseases.

[6] Chen Y.H., Keiser M.S., Davidson B.L. (2018). Viral vectors for gene transfer. Curr. Protoc. Mouse Biol..

[7] Li C., Samulski R.J. (2020). Engineering adeno-associated virus vectors for gene therapy. Nat. Rev. Genet..

[8] Schmidt M., Govindasamy L., Afione S., Kaludov N., Agbandje-McKenna M., Chiorini J.A. (2008). Molecular characterization of the heparin-dependent transduction domain on the capsid of a novel adeno-associated virus isolate, AAV(VR-942). J. Virol..

[9] Weinmann J., Grimm D. (2017). Next-generation AAV vectors for clinical use: an ever-accelerating race. Virus Genes.

[10] Berry G.E., Asokan A. (2016). Cellular transduction mechanisms of adeno-associated viral vectors. Curr. Opin. Virol..

[11] Pattali R., Mou Y., Li X.-J. (2019). AAV9 Vector: a Novel modality in gene therapy for spinal muscular atrophy. Gene Ther..

[12] Pei X., Shao W., Xing A., Askew C., Chen X., Cui C., Abajas Y.L., Gerber D.A., Merricks E.P., Nichols T.C. (2020). Development of AAV variants with human hepatocyte tropism and neutralizing antibody escape capacity. Mol. Ther. Methods Clin. Dev..

[13] Davidson B.L., Stein C.S., Heth J.A., Martins I., Kotin R.M., Derksen T.A., Zabner J., Ghodsi A., Chiorini J.A., Ghodsi A. (2000). Recombinant adeno-associated virus type 2, 4, and 5 vectors: transduction of variant cell types and regions in the mammalian central nervous system. Proc. Natl. Acad. Sci. U S A.

[14] Kantor B., Bailey R.M., Wimberly K., Kalburgi S.N., Gray S.J. (2014). Methods for gene transfer to the central nervous system. Adv. Genet..

[15] Wang D., Tai P.W.L., Gao G. (2019). Adeno-associated virus vector as a platform for gene therapy delivery. Nat. Rev. Drug Discov..

[16] Ferrari F.K., Samulski T., Shenk T., Samulski R.J. (1996). Second-strand synthesis is a rate-limiting step for efficient transduction by recombinant adeno-associated virus vectors. J. Virol..

[17] Nakai H., Montini E., Fuess S., Storm T.A., Grompe M., Kay M.A. (2003). AAV serotype 2 vectors preferentially integrate into active genes in mice. Nat. Genet..

[18] Nakai H., Montini E., Fuess S., Storm T.A., Meuse L., Finegold M., Grompe M., Kay M.A. (2003). Helper-independent and AAV-ITR-independent chromosomal integration of double-stranded linear DNA vectors in mice. Mol. Ther..

[19] Kotin R.M., Siniscalco M., Samulski R.J., Zhu X.D., Hunter L., Laughlin C.A., McLaughlin S., Muzyczka N., Rocchi M., Berns K.I. (1990). Site-specific integration by adeno-associated virus. Proc. Natl. Acad. Sci. U S A.

[20] Samulski R.J., Zhu X., Xiao X., Brook J.D., Housman D.E., Epstein N., Hunter L.A. (1991). Targeted integration of adeno-associated virus (AAV) into human chromosome 19. EMBO. J..

[21] Hüser D., Gogol-Döring A., Lutter T., Weger S., Winter K., Hammer E.M., Cathomen T., Reinert K., Heilbronn R. (2010). Integration preferences of wildtype AAV-2 for consensus rep-binding sites at numerous loci in the human genome. PLoS. Pathog..

[22] McCarty D.M., Young S.M., Samulski R.J. (2004). Integration of adeno-associated virus (AAV) and recombinant AAV vectors. Annu. Rev. Genet..

[23] Nowrouzi A., Penaud-Budloo M., Kaeppel C., Appelt U., Le Guiner C., Moullier P., von Kalle C., Snyder R.O., Schmidt M. (2012). Integration frequency and intermolecular recombination of rAAV vectors in non-human primate skeletal muscle and liver. Mol. Ther..

[24] Chandler R.J., LaFave M.C., Varshney G.K., Burgess S.M., Venditti C.P. (2016). Genotoxicity in mice following AAV gene delivery: a safety concern for human gene therapy?. Mol. Ther..

[25] Chandler R.J., Sands M.S., Venditti C.P. (2017). Recombinant adeno-associated viral integration and genotoxicity: insights from animal models. Hum. Gene Ther..

[26] de Jong Y.P., Herzog R.W. (2021). Liver gene therapy and hepatocellular carcinoma: a complex web. Mol. Ther..

[27] Gil-Farina I., Fronza R., Kaeppel C., Lopez-Franco E., Ferreira V., D'Avola D., Benito A., Prieto J., Petry H., Gonzalez-Aseguinolaza G., Schmidt M. (2016). Recombinant AAV integration is not associated with hepatic genotoxicity in nonhuman primates and patients. Mol. Ther..

[28] EMA (2012). Glybera [Press release]. https://www.ema.europa.eu/en/medicines/human/EPAR/glybera.

[29] EMA (2018). New gene therapy for rare inherited disorder causing vision loss recommended for approval [Press release]. https://www.ema.europa.eu/en/news/new-gene-therapy-rare-inherited-disorder-causing-vision-loss-recommended-approval.

[30] FDA (2017). FDA approves novel gene therapy to treat patients with a rare form of inherited vision loss [Press release]. https://www.fda.gov/news-events/press-announcements/fda-approves-novel-gene-therapy-treat-patients-rare-form-inherited-vision-loss.

[31] FDA (2019). FDA approves innovative gene therapy to treat pediatric patients with spinal muscular atrophy, a rare disease and leading genetic cause of infant mortality [Press release]. https://www.fda.gov/news-events/press-announcements/fda-approves-innovative-gene-therapy-treat-pediatric-patients-spinal-muscular-atrophy-rare-disease.

[32] EMA (2020). New gene therapy to treat spinal muscular atrophy [Press release]. https://www.ema.europa.eu/en/documents/press-release/new-gene-therapy-treat-spinal-muscular-atrophy_en.pdf.

[33] Mendell J.R., Al-Zaidy S.A., Rodino-Klapac L.R., Goodspeed K., Gray S.J., Kay C.N., Boye S.L., Boye S.E., George L.A., Salabarria S. (2021). Current clinical applications of in vivo gene therapy with AAVs. Mol. Ther..

[34] Kuzmin D.A., Shutova M.V., Johnston N.R., Smith O.P., Fedorin V.V., Kukushkin Y.S., van der Loo J.C.M., Johnstone E.C. (2021). The clinical landscape for AAV gene therapies. Nat. Rev. Drug Discov..

[35] Rutledge E.A., Russell D.W. (1997). Adeno-associated virus vector integration junctions. J. Virol..

[36] Yang C.C., Xiao X., Zhu X., Ansardi D.C., Epstein N.D., Frey M.R., Matera A.G., Samulski R.J. (1997). Cellular recombination pathways and viral terminal repeat hairpin structures are sufficient for adeno-associated virus integration in vivo and in vitro. J. Virol..

[37] Nakai H., Iwaki Y., Kay M.A., Couto L.B. (1999). Isolation of recombinant adeno-associated virus vector-cellular DNA junctions from mouse liver. J. Virol..

[38] Nakai H., Yant S.R., Storm T.A., Fuess S., Meuse L., Kay M.A. (2001). Extrachromosomal recombinant adeno-associated virus vector genomes are primarily responsible for stable liver transduction in vivo. J. Virol..

[39] Nakai H., Wu X., Fuess S., Storm T.A., Munroe D., Montini E., Burgess S.M., Grompe M., Kay M.A. (2005). Large-scale molecular characterization of adeno-associated virus vector integration in mouse liver. J. Virol..

[40] Miller D.G., Trobridge G.D., Petek L.M., Jacobs M.A., Kaul R., Russell D.W. (2005). Large-scale analysis of adeno-associated virus vector integration sites in normal human cells. J. Virol..

[41] Miller D.G., Petek L.M., Russell D.W. (2004). Adeno-associated virus vectors integrate at chromosome breakage sites. Nat. Genet..

[42] Donsante A., Vogler C., Muzyczka N., Crawford J.M., Barker J., Flotte T., Campbell-Thompson M., Daly T., Sands M.S., DonsAnte A. (2001). Observed incidence of tumorigenesis in long-term rodent studies of rAAV vectors. Gene Ther..

[43] Sands M.S., Birkenmeier E.H. (1993). A single-base-pair deletion in the beta-glucuronidase gene accounts for the phenotype of murine mucopolysaccharidosis type VII. Proc. Natl. Acad. Sci. U S A.

[44] Daly T.M., Ohlemiller K.K., Roberts M.S., Vogler C.A., Sands M.S. (2001). Prevention of systemic clinical disease in MPS VII mice following AAV-mediated neonatal gene transfer. Gene Ther..

[45] Daly T.M., Okuyama T., Vogler C., Haskins M.E., Muzyczka N., Sands M.S. (1999). Neonatal intramuscular injection with recombinant adeno-associated virus results in prolonged beta-glucuronidase expression in situ and correction of liver pathology in mucopolysaccharidosis type VII mice. Hum. Gene Ther..

[46] Donsante A., Miller D.G., Li Y., Vogler C., Brunt E.M., Russell D.W., Sands M.S. (2007). AAV vector integration sites in mouse hepatocellular carcinoma. Science.

[47] Bell P., Wang L., Lebherz C., Flieder D.B., Bove M.S., Wu D., Gao G.P., Wilson J.M., Wivel N.A. (2005). No evidence for tumorigenesis of AAV vectors in a large-scale study in mice. Mol. Ther..

[48] Bell P., Moscioni A.D., McCarter R.J., Wu D., Gao G., Hoang A., Sanmiguel J.C., Sun X., Wivel N.A., Raper S.E. (2006). Analysis of tumors arising in male B6C3F1 mice with and without AAV vector delivery to liver. Mol. Ther..

[49] Zhong L., Malani N., Li M., Brady T., Xie J., Bell P., Li S., Jones H., Wilson J.M., Flotte T.R. (2013). Recombinant adeno-associated virus integration sites in murine liver after ornithine transcarbamylase gene correction. Hum. Gene Ther..

[50] Reiss J., Hahnewald R. (2011). Molybdenum cofactor deficiency: mutations in GPHN, MOCS1, and MOCS2. Hum. Mutat..

[51] Embury J.E., Frost S., Charron C.E., Madrigal E., Perera O.P., Poirier A., Zori A.G., Carmical R., Flotte T.R., Laipis P.J. (2008). Hepatitis Virus Protein X-Phenylalanine Hydroxylase fusion proteins identified in PKU mice treated with AAV-WPRE vectors. Gene Ther. Mol. Bio..

[52] Li H., Malani N., Hamilton S.R., Schlachterman A., Bussadori G., Edmonson S.E., Shah R., Arruda V.R., Mingozzi F., Fraser Wright J. (2011). Assessing the potential for AAV vector genotoxicity in a murine model. Blood.

[53] Wang P.R., Xu M., Toffanin S., Li Y., Llovet J.M., Russell D.W. (2012). Induction of hepatocellular carcinoma by in vivo gene targeting. Proc. Natl. Acad. Sci. U S A.

[54] Rosas L.E., Grieves J.L., Zaraspe K., La Perle K.M., Fu H., McCarty D.M. (2012). Patterns of scAAV vector insertion associated with oncogenic events in a mouse model for genotoxicity. Mol. Ther..

[55] Kao C.Y., Yang S.J., Tao M.H., Jeng Y.M., Yu I.S., Lin S.W. (2013). Incorporation of the factor IX Padua mutation into FIX-Triple improves clotting activity in vitro and in vivo. Thromb. Haemost..

[56] Chandler R.J., LaFave M.C., Varshney G.K., Trivedi N.S., Carrillo-Carrasco N., Senac J.S., Wu W., Hoffmann V., Elkahloun A.G., Burgess S.M., Venditti C.P. (2015). Vector design influences hepatic genotoxicity after adeno-associated virus gene therapy. J. Clin. Invest..

[57] Walia J.S., Altaleb N., Bello A., Kruck C., LaFave M.C., Varshney G.K., Burgess S.M., Chowdhury B., Hurlbut D., Hemming R. (2015). Long-term correction of Sandhoff disease following intravenous delivery of rAAV9 to mouse neonates. Mol. Ther..

[58] Kang H.R., Gjorgjieva M., Smith S.N., Brooks E.D., Chen Z., Burgess S.M., Chandler R.J., Waskowicz L.R., Grady K.M., Li S. (2019). Pathogenesis of hepatic tumors following gene therapy in murine and canine models of glycogen storage disease. Mol. Ther. Methods Clin. Dev..

[59] Dalwadi D.A., Torrens L., Abril-Fornaguera J., Pinyol R., Willoughby C., Posey J., Llovet J.M., Lanciault C., Russell D.W., Grompe M., Naugler W.E. (2021). Liver injury increases the incidence of HCC following AAV gene therapy in mice. Mol. Ther..

[60] Li Y., Miller C.A., Shea L.K., Jiang X., Guzman M.A., Chandler R.J., Ramakrishnan S.M., Smith S.N., Venditti C.P., Vogler C.A. (2021). Enhanced efficacy and increased long-term toxicity of CNS-directed, AAV-based combination therapy for Krabbe disease. Mol. Ther..

[61] Dupuy A.J., Rogers L.M., Kim J., Nannapaneni K., Starr T.K., Liu P., Largaespada D.A., Scheetz T.E., Jenkins N.A., Copeland N.G. (2009). A modified sleeping beauty transposon system that can be used to model a wide variety of human cancers in mice. Cancer Res..

[62] Riordan J.D., Keng V.W., Tschida B.R., Scheetz T.E., Bell J.B., Podetz-Pedersen K.M., Moser C.D., Copeland N.G., Jenkins N.A., Roberts L.R. (2013). Identification of rtl1, a retrotransposon-derived imprinted gene, as a novel driver of hepatocarcinogenesis. PLoS. Genet..

[63] Ranzani M., Cesana D., Bartholomae C.C., Sanvito F., Pala M., Benedicenti F., Gallina P., Sergi L.S., Merella S., Bulfone A. (2013). Lentiviral vector-based insertional mutagenesis identifies genes associated with liver cancer. Nat. Methods.

[64] Hu Y., Yan W., Jin Z., Li Q.Y., Yang X., Zhu A.K., Shan W., Tan B.Q., Du Q., Dong R. (2021). LncRNA MEG8 plays an oncogenic role in hepatocellular carcinoma progression through miR-367-3p/14-3-3ζ/TGFβR1 axis. Neoplasma.

[65] Cataldi M.P., McCarty D.M. (2010). Differential effects of DNA double-strand break repair pathways on single-strand and self-complementary adeno-associated virus vector genomes. J. Virol..

[66] Knudson A.G. (1971). Mutation and cancer: statistical study of retinoblastoma. Proc. Natl. Acad. Sci. U S A.

[67] Colella P., Ronzitti G., Mingozzi F. (2018). Emerging issues in AAV-mediated in vivo gene therapy. Mol. Ther. Methods Clin. Dev..

[68] Ferla R., Alliegro M., Dell'Anno M., Nusco E., Cullen J.M., Smith S.N., Wolfsberg T.G., O'Donnell P., Wang P., Nguyen A.D. (2021). Low incidence of hepatocellular carcinoma in mice and cats treated with systemic adeno-associated viral vectors. Mol. Ther. Methods Clin. Dev..

[69] Li C., Yu S., Zhong X., Wu J., Li X. (2012). Transcriptome comparison between fetal and adult mouse livers: implications for circadian clock mechanisms. PLoS One.

[70] Kim N.N., Parker R.M., Weinbauer G.F., Remick A.K., Steinbach T. (2017). Points to consider in designing and conducting juvenile toxicology studies. Int. J. Toxicol..

[71] Gauttier V., Pichard V., Aubert D., Kaeppel C., Schmidt M., Ferry N., Conchon S. (2013). No tumour-initiating risk associated with scAAV transduction in newborn rat liver. Gene Ther..

[72] Schmidt E.V., Christoph G., Zeller R., Leder P. (1990). The cytomegalovirus enhancer: a pan-active control element in transgenic mice. Mol. Cell. Biol..

[73] Kay M.A., Li Q., Liu T.J., Leland F., Toman C., Finegold M., Woo S.L.C. (1992). Hepatic gene therapy: persistent expression of human alpha 1-antitrypsin in mice after direct gene delivery in vivo. Hum. Gene Ther..

[74] Mattar C.N.Z., Gil-Farina I., Rosales C., Johana N., Tan Y.Y.W., McIntosh J., Kaeppel C., Waddington S.N., Biswas A., Choolani M. (2017). In utero transfer of adeno-associated viral vectors produces long-term factor IX levels in a cynomolgus macaque model. Mol. Ther..

[75] Spronck L., de Haan M., Heijink L., Twisk J., Ferreira V., van Deventer S. (2020). Assessment of vector integration of AAV5-hFIX in mice and non-human primates indicates No association with tumorigenic risk [Abstract]. Res. Pract. Thromb. Haemost..

[76] Sullivan L., Zahn M., Gil Farina I., Kasprzyk T., O'Neill C.A., Eggan K., Zoog S.J., Veres G., Schmidt M., Vettermann C. (2021). Rare genomic integrations of AAV5-hFVIII-SQ occur without evidence of clonal activation or gene-specific targeting. Mol. Ther..

[77] Mattar C.N., Nathwani A.C., Waddington S.N., Dighe N., Kaeppel C., Nowrouzi A., McIntosh J., Johana N.B., Ogden B., Fisk N.M. (2011). Stable human FIX expression after 0.9G intrauterine gene transfer of self-complementary adeno-associated viral vector 5 and 8 in macaques. Mol. Ther..

[78] Niemeyer G.P., Herzog R.W., Mount J., Arruda V.R., Tillson D.M., Hathcock J., van Ginkel F.W., High K.A., Lothrop C.D. (2009). Long-term correction of inhibitor-prone hemophilia B dogs treated with liver-directed AAV2-mediated factor IX gene therapy. Blood.

[79] Nguyen G.N., Everett J.K., Kafle S., Roche A.M., Raymond H.E., Leiby J., Wood C., Assenmacher C.A., Merricks E.P., Long C.T. (2021). A long-term study of AAV gene therapy in dogs with hemophilia A identifies clonal expansions of transduced liver cells. Nat. Biotechnol..

[80] Batty P., Sihn C.R., Ishida J., Mo A.M., Yates B., Brown C., Harpell L., Pender A., Russell C., Sardo Infirri S. (2020). Long-term vector genome outcomes and immunogenicity of AAV FVIII gene transfer in the hemophilia A dog model [abstract]. Res. Pract. Thromb. Haemost..

[81] Batty P., Fong S., Franco M., Gil-Farina I., Mo A.M., Harpell L., Hough C., Hurlbut D., Pender A., Sardo Infirri S. (2020). Frequency, location and nature of AAV vector insertions after long-term follow up of FVIII transgene delivery in a Hemophilia A dog model [abstract]. Res. Pract. Thromb. Haemost..

[82] Nathwani A.C., Rosales C., McIntosh J., Rastegarlari G., Nathwani D., Raj D., Nawathe S., Waddington S.N., Bronson R., Jackson S. (2011). Long-term safety and efficacy following systemic administration of a self-complementary AAV vector encoding human FIX pseudotyped with serotype 5 and 8 capsid proteins. Mol. Ther..

[83] Li T., Huang J., Jiang Y., Zeng Y., He F., Zhang M.Q., Han Z., Zhang X. (2009). Multi-stage analysis of gene expression and transcription regulation in C57/B6 mouse liver development. Genomics.

[84] Sabatino D.E., Lange A.M., Altynova E.S., Sarkar R., Zhou S., Merricks E.P., Franck H.G., Nichols T.C., Arruda V.R., Kazazian Jr H.H. (2011). Efficacy and safety of long-term prophylaxis in severe hemophilia A dogs following liver gene therapy using AAV vectors. Mol. Ther..

[85] Venditti C.P. (2021). Safety questions for AAV gene therapy. Nat. Biotechnol..

[86] Arruda V.R., Schuettrumpf J., Herzog R.W., Nichols T.C., Robinson N., Lotfi Y., Mingozzi F., Xiao W., Couto L.B., High K.A. (2004). Safety and efficacy of factor IX gene transfer to skeletal muscle in murine and canine hemophilia B models by adeno-associated viral vector serotype 1. Blood.

[87] Nichols T.C., Whitford M.H., Arruda V.R., Stedman H.H., Kay M.A., High K.A. (2015). Translational data from adeno-associated virus-mediated gene therapy of hemophilia B in dogs. Hum. Gene Ther. Clin. Dev..

[88] Dalwadi D.A., Calabria A., Tiyaboonchai A., Posey J., Naugler W.E., Montini E., Grompe M. (2021). AAV integration in human hepatocytes. Mol. Ther..

[89] George L.A., Ragni M.V., Rasko J.E.J., Raffini L.J., Samelson-Jones B.J., Ozelo M., Hazbon M., Runowski A.R., Wellman J.A., Wachtel K. (2020). Long-term follow-up of the first in human intravascular delivery of AAV for gene transfer: AAV2-hFIX16 for severe hemophilia B. Mol. Ther..

[90] Nathwani A.C., Reiss U., Tuddenham E., Chowdary P., McIntosh J., Riddell A., Pie J., Mahlangu J.N., Recht M., Shen Y.-M. (2018). Adeno-associated mediated gene transfer for hemophilia B:8 year follow up and impact of removing "empty viral particles" on safety and efficacy of gene transfer. Blood.

[91] Pasi K.J., Rangarajan S., Mitchell N., Lester W., Symington E., Madan B., Laffan M., Russell C.B., Li M., Pierce G.F., Wong W.Y. (2020). Multiyear follow-up of AAV5-hFVIII-SQ gene therapy for hemophilia A. N. Engl. J. Med..

[92] Rangarajan S., Walsh L., Lester W., Perry D., Madan B., Laffan M., Yu H., Vettermann C., Pierce G.F., Wong W.Y., Pasi K.J. (2017). AAV5–Factor VIII gene transfer in severe hemophilia A. N. Engl. J. Med..

[93] George L.A., Monahan P.E., Eyster M.E., Sullivan S.K., Ragni M.V., Croteau S.E., Rasko J.E.J., Recht M., Samelson-Jones B.J., MacDougall A. (2021). Multiyear factor VIII expression after AAV gene transfer for hemophilia A. N. Engl. J. Med..

[94] Srivastava A., Carter B.J. (2017). AAV infection: protection from cancer. Hum. Gene Ther..

[95] Kaeppel C., Beattie S.G., Fronza R., van Logtenstein R., Salmon F., Schmidt S., Wolf S., Nowrouzi A., Glimm H., von Kalle C. (2013). A largely random AAV integration profile after LPLD gene therapy. Nat. Med..

[96] Pañeda A., Lopez-Franco E., Kaeppel C., Unzu C., Gil-Royo A.G., D'Avola D., Beattie S.G., Olagüe C., Ferrero R., Sampedro A. (2013). Safety and liver transduction efficacy of rAAV5-cohPBGD in nonhuman primates: a potential therapy for acute intermittent porphyria. Hum. Gene Ther..

[97] uniQure (2020). uniQure announces clinical update on Hemophilia B gene therapy program [Press release]. https://www.globenewswire.com/news-release/2020/12/21/2148517/0/en/uniQure-Announces-Clinical-Update-on-Hemophilia-B-Gene-Therapy-Program.html.

[98] uniQure (2021). uniQure announces FDA removes clinical hold on Hemophilia B gene therapy program [Press release]. https://www.globenewswire.com/news-release/2021/04/26/2216691/0/en/uniQure-Announces-FDA-Removes-Clinical-Hold-on-Hemophilia-B-Gene-Therapy-Program.html.

[99] uniQure (2022). uniQure has built a potential first- and best-in-class hemophilia B gene therapy program [Press release]. http://uniqure.com/gene-therapy/hemophilia.php.

[100] Schmidt M.R., Foster G., Coppens M., Thomsen H., Cooper D., Dolmetsch R.K., Sawyer E., Heijink L., Pipe S. (2021). Liver safety case report from the phase 3 HOPE-B gene therapy trial in adults with hemophilia B [abstract]. Res. Pract. Thromb. Haemost..

[101] uniQure (2021). uniQure announces findings from reported case of Hepatocellular Carcinoma (HCC) in Hemophilia B gene therapy program [Press release]. https://tools.eurolandir.com/tools/Pressreleases/GetPressRelease/?ID=3890956&lang=en-GB&companycode=nl-qure&v=.

[102] Bushman F.D., Cantu A., Everett J., Sabatino D., Berry C. (2021). Challenges in estimating numbers of vectors integrated in gene-modified cells using DNA sequence information. Mol. Ther..

[103] Schmidt M., Schwarzwaelder K., Bartholomae C., Zaoui K., Ball C., Pilz I., Braun S., Glimm H., von Kalle C. (2007). High-resolution insertion-site analysis by linear amplification-mediated PCR (LAM-PCR). Nat. Methods.

[104] Sherman E., Nobles C., Berry C.C., Six E., Wu Y., Dryga A., Malani N., Male F., Reddy S., Bailey A. (2017). INSPIIRED: a pipeline for quantitative analysis of sites of new DNA integration in cellular genomes. Mol. Ther. Methods Clin. Dev..

[105] Berry C.C., Nobles C., Six E., Wu Y., Malani N., Sherman E., Dryga A., Everett J.K., Male F., Bailey A. (2017). INSPIIRED: quantification and visualization tools for analyzing integration site distributions. Mol. Ther. Methods Clin. Dev..

[106] Locatelli F., Thompson A.A., Kwiatkowski J.L., Porter J.B., Thrasher A.J., Hongeng S., Sauer M.G., Thuret I., Lal A., Algeri M. (2022). Betibeglogene Autotemcel gene therapy for non-β^0^/β^0^ genotype β-Thalassemia. N. Engl. J. Med..

[107] Berry C.C., Gillet N.A., Melamed A., Gormley N., Bangham C.R.M., Bushman F.D. (2012). Estimating abundances of retroviral insertion sites from DNA fragment length data. J. Bioinform..

[108] Senís E., Mosteiro L., Wilkening S., Wiedtke E., Nowrouzi A., Afzal S., Fronza R., Landerer H., Abad M., Niopek D. (2018). AAV vector-mediated in vivo reprogramming into pluripotency. Nat. Commun..

[109] Tai P.W.L., Xie J., Fong K., Seetin M., Heiner C., Su Q., Weiand M., Wilmot D., Zapp M.L., Gao G. (2018). Adeno-associated virus genome population sequencing achieves full vector genome resolution and reveals human-vector chimeras. Mol. Ther. Methods Clin. Dev..

[110] Zhang J., Yu X., Herzog R.W., Samulski R.J., Xiao W. (2021). Flies in the ointment: AAV vector preparations and tumor risk. Mol. Ther..

[111] Dash S., Kinney N.A., Varghese R.T., Garner H.R., Feng W.-C., Anandakrishnan R. (2019). Differentiating between cancer and normal tissue samples using multi-hit combinations of genetic mutations. Sci. Rep..

[112] Guerin K., Rego M., Bourges D., Ersing I., Haery L., Harten DeMaio K., Sanders E., Tasissa M., Kostman M., Tillgren M. (2020). A novel next-generation sequencing and analysis platform to assess the identity of recombinant adeno-associated viral preparations from viral DNA extracts. Hum. Gene Ther..

[113] Chao A., Chazdon R.L., Colwell R.K., Shen T.J. (2006). Abundance-based similarity indices and their estimation when there are unseen species in samples. Biometrics.

[114] Cesana D., Calabria A., Rudilosso L., Gallina P., Benedicenti F., Spinozzi G., Schiroli G., Magnani A., Acquati S., Fumagalli F. (2021). Retrieval of vector integration sites from cell-free DNA. Nat. Med*.*.

[115] Cesana D., Calabria A., Rudilosso L., Gallina P., Benedicenti F., Spinozzi G., Volpin M., Schiroli G., Magnani A., Pouzolles M. (2021). Liquid-biopsy-integration-site sequencing allows safety studies and longitudinal monitoring of vector integration sites in LV and AAV-based in-vivo GT applications. Mol. Ther..

[116] Bijlani S., Pang K.M., Sivanandam V., Singh A., Chatterjee S. (2022). The role of recombinant AAV in precise genome editing. Front. Genome Ed..

[117] Berry C., Hannenhalli S., Leipzig J., Bushman F.D. (2006). Selection of target sites for mobile DNA integration in the human genome. PLoS Comput. Biol..

[118] Braun C.J., Boztug K., Paruzynski A., Witzel M., Schwarzer A., Rothe M., Modlich U., Beier R., Göhring G., Steinemann D. (2014). Gene therapy for Wiskott-Aldrich syndrome--long-term efficacy and genotoxicity. Sci. Transl. Med..

[119] Bushman F.D. (2020). Retroviral insertional mutagenesis in humans: evidence for four genetic mechanisms promoting expansion of cell clones. Mol. Ther..

[120] Hacein-Bey-Abina S., Garrigue A., Wang G.P., Soulier J., Lim A., Morillon E., Clappier E., Caccavelli L., Delabesse E., Beldjord K. (2008). Insertional oncogenesis in 4 patients after retrovirus-mediated gene therapy of SCID-X1. J. Clin. Invest..

[121] Hacein-Bey-Abina S., von Kalle C., Schmidt M., Le Deist F., Wulffraat N., McIntyre E., Radford I., Villeval J.L., Fraser C.C., Cavazzana-Calvo M., Fischer A. (2003). A serious adverse event after successful gene therapy for X-linked severe combined immunodeficiency. N. Engl. J. Med..

[122] Hacein-Bey-Abina S., Von Kalle C., Schmidt M., McCormack M.P., Wulffraat N., Leboulch P., Lim A., Osborne C.S., Pawliuk R., Morillon E. (2003). LMO2-associated clonal T cell proliferation in two patients after gene therapy for SCID-X1. Science.

[123] Montini E., Cesana D., Schmidt M., Sanvito F., Bartholomae C.C., Ranzani M., Benedicenti F., Sergi L.S., Ambrosi A., Ponzoni M. (2009). The genotoxic potential of retroviral vectors is strongly modulated by vector design and integration site selection in a mouse model of HSC gene therapy. J. Clin. Invest..

[124] Ott M.G., Schmidt M., Schwarzwaelder K., Stein S., Siler U., Koehl U., Glimm H., Kühlcke K., Schilz A., Kunkel H. (2006). Correction of X-linked chronic granulomatous disease by gene therapy, augmented by insertional activation of MDS1-EVI1, PRDM16 or SETBP1. Nat. Med..

[125] EMA (2021). Strimvelis® (autologous CD34+ enriched cell fraction that contains CD34+ cells transduced with retroviral vector that encodes for the human adenosine deaminase [ADA] cDNA sequence): first case of lymphoid T cell leukaemia after insertional oncogenesis [Press release]. https://www.ema.europa.eu/en/documents/dhpc/direct-healthcare-professional-communication-dhpc-strimvelis-first-case-lymphoid-t-cell-leukaemia_en.pdf.

[126] Cavazzana M., Bushman F.D., Miccio A., André-Schmutz I., Six E. (2019). Gene therapy targeting haematopoietic stem cells for inherited diseases: progress and challenges. Nat. Rev. Drug Discov..

[127] Deichmann A., Hacein-Bey-Abina S., Schmidt M., Garrigue A., Brugman M.H., Hu J., Glimm H., Gyay G., Prum B., Fraser C.C. (2007). Vector integration is nonrandom and clustered and influences the fate of lymphopoiesis in SCID-X1 gene therapy. J. Clin. Invest..

[128] Howe S.J., Mansour M.R., Schwarzwaelder K., Bartholomae C., Hubank M., Kempski H., Brugman M.H., Pike-Overzet K., Chatters S.J., de Ridder D. (2008). Insertional mutagenesis combined with acquired somatic mutations causes leukemogenesis following gene therapy of SCID-X1 patients. J. Clin. Invest..

[129] Stein S., Ott M.G., Schultze-Strasser S., Jauch A., Burwinkel B., Kinner A., Schmidt M., Krämer A., Schwäble J., Glimm H. (2010). Genomic instability and myelodysplasia with monosomy 7 consequent to EVI1 activation after gene therapy for chronic granulomatous disease. Nat. Med..

[130] Klein C., Nguyen D., Liu C.H., Mizoguchi A., Bhan A.K., Miki H., Takenawa T., Rosen F.S., Alt F.W., Mulligan R.C., Snapper S.B. (2003). Gene therapy for Wiskott-Aldrich syndrome: rescue of T-cell signaling and amelioration of colitis upon transplantation of retrovirally transduced hematopoietic stem cells in mice. Blood.

[131] Lo Y.M.D., Zhang J., Leung T.N., Lau T.K., Chang A.M., Hjelm N.M. (1999). Rapid clearance of fetal DNA from maternal plasma. Am. J. Hum. Genet..

[132] Otsu M., Anderson S.M., Bodine D.M., Puck J.M., O'Shea J.J., Candotti F. (2000). Lymphoid development and function in X-linked severe combined immunodeficiency mice after stem cell gene therapy. Mol. Ther..

[133] Stein S., Scholz S., Schwäble J., Sadat M.A., Modlich U., Schultze-Strasser S., Diaz M., Chen-Wichmann L., Müller-Kuller U., Brendel C. (2013). From bench to bedside: preclinical evaluation of a self-inactivating gammaretroviral vector for the gene therapy of X-linked chronic granulomatous disease. Hum. Gene Ther. Clin. Dev..

[134] Strom T.S., Gabbard W., Kelly P.F., Cunningham J.M., Nienhuis A.W. (2003). Functional correction of T cells derived from patients with the Wiskott-Aldrich syndrome (WAS) by transduction with an oncoretroviral vector encoding the WAS protein. Gene Ther..

[135] Li Z., Düllmann J., Schiedlmeier B., Schmidt M., von Kalle C., Meyer J., Forster M., Stocking C., Wahlers A., Frank O. (2002). Murine leukemia induced by retroviral gene marking. Science.

[136] Modlich U., Schambach A., Brugman M.H., Wicke D.C., Knoess S., Li Z., Maetzig T., Rudolph C., Schlegelberger B., Baum C. (2008). Leukemia induction after a single retroviral vector insertion in Evi1 or Prdm16. Leukemia.

[137] Modlich U., Kustikova O.S., Schmidt M., Rudolph C., Meyer J., Li Z., Kamino K., von Neuhoff N., Schlegelberger B., Kuehlcke K. (2005). Leukemias following retroviral transfer of multidrug resistance 1 (MDR1) are driven by combinatorial insertional mutagenesis. Blood.

[138] Will E., Bailey J., Schuesler T., Modlich U., Balcik B., Burzynski B., Witte D., Layh-Schmitt G., Rudolph C., Schlegelberger B. (2007). Importance of murine study design for testing toxicity of retroviral vectors in support of phase I trials. Mol. Ther..

[139] Modlich U., Bohne J., Schmidt M., von Kalle C., Knöss S., Schambach A., Baum C. (2006). Cell-culture assays reveal the importance of retroviral vector design for insertional genotoxicity. Blood.

[140] Modlich U., Navarro S., Zychlinski D., Maetzig T., Knoess S., Brugman M.H., Schambach A., Charrier S., Galy A., Thrasher A.J. (2009). Insertional transformation of hematopoietic cells by self-inactivating lentiviral and gammaretroviral vectors. Mol. Ther..

[141] Cesana D., Sgualdino J., Rudilosso L., Merella S., Naldini L., Montini E. (2012). Whole transcriptome characterization of aberrant splicing events induced by lentiviral vector integrations. J. Clin. Invest..

[142] Montini E., Cesana D., Schmidt M., Sanvito F., Ponzoni M., Bartholomae C., Sergi L.S., Benedicenti F., Ambrosi A., Di Serio C. (2006). Hematopoietic stem cell gene transfer in a tumor-prone mouse model uncovers low genotoxicity of lentiviral vector integration. Nat. Biotechnol..

[143] Schwarzer A., Talbot S.R., Selich A., Morgan M., Schott J.W., Dittrich-Breiholz O., Bastone A.L., Weigel B., Ha T.C., Dziadek V. (2021). Predicting genotoxicity of viral vectors for stem cell gene therapy using gene expression-based machine learning. Mol. Ther..

[144] Zychlinski D., Schambach A., Modlich U., Maetzig T., Meyer J., Grassman E., Mishra A., Baum C. (2008). Physiological promoters reduce the genotoxic risk of integrating gene vectors. Mol. Ther..

[145] Aiuti A., Biasco L., Scaramuzza S., Ferrua F., Cicalese M.P., Baricordi C., Dionisio F., Calabria A., Giannelli S., Castiello M.C. (2013). Lentiviral hematopoietic stem cell gene therapy in patients with Wiskott-Aldrich syndrome. Science.

[146] Biffi G., Tannahill D., McCafferty J., Balasubramanian S. (2013). Quantitative visualization of DNA G-quadruplex structures in human cells. Nat. Chem..

[147] Cartier N., Hacein-Bey-Abina S., Bartholomae C.C., Veres G., Schmidt M., Kutschera I., Vidaud M., Abel U., Dal-Cortivo L., Caccavelli L. (2009). Hematopoietic stem cell gene therapy with a lentiviral vector in X-linked adrenoleukodystrophy. Science.

[148] Cavazzana-Calvo M., Payen E., Negre O., Wang G., Hehir K., Fusil F., Down J., Denaro M., Brady T., Westerman K. (2010). Transfusion independence and HMGA2 activation after gene therapy of human β-thalassaemia. Nature.

[149] Hacein-Bey Abina S., Gaspar H.B., Blondeau J., Caccavelli L., Charrier S., Buckland K., Picard C., Six E., Himoudi N., Gilmour K. (2015). Outcomes following gene therapy in patients with severe Wiskott-Aldrich syndrome. JAMA.

[150] Hacein-Bey-Abina S., Pai S.Y., Gaspar H.B., Armant M., Berry C.C., Blanche S., Bleesing J., Blondeau J., de Boer H., Buckland K.F. (2014). A modified γ-retrovirus vector for X-linked severe combined immunodeficiency. N. Engl. J. Med..

[151] Naldini L. (2011). Ex vivo gene transfer and correction for cell-based therapies. Nat. Rev. Genet..

[152] Cesana D., Ranzani M., Volpin M., Bartholomae C., Duros C., Artus A., Merella S., Benedicenti F., Sergi Sergi L., Sanvito F. (2014). Uncovering and dissecting the genotoxicity of self-inactivating lentiviral vectors *in vivo*. Mol. Ther..

[153] Kay M.A., Baley P., Rothenberg S., Leland F., Fleming L., Ponder K.P., Liu T., Finegold M., Darlington G., Pokorny W. (1992). Expression of human alpha 1-antitrypsin in dogs after autologous transplantation of retroviral transduced hepatocytes. Proc. Natl. Acad. Sci. U S A.

[154] Logan G.J., Dane A.P., Hallwirth C.V., Smyth C.M., Wilkie E.E., Amaya A.K., Zhu E., Khandekar N., Ginn S.L., Liao S.H.Y. (2017). Identification of liver-specific enhancer-promoter activity in the 3' untranslated region of the wild-type AAV2 genome. Nat. Genet..

[155] Novartis (2022). Novartis data again demonstrate age-appropriate development when Zolgensma is used presymptomatically, and post-hoc data reveal SMA Type 1 patients could speak, swallow and maintain airway protection [Press release]. https://www.novartis.com/news/media-releases/novartis-data-again-demonstrate-age-appropriate-development-when-zolgensma-used-presymptomatically-and-post-hoc-data-reveal-sma-type-1-patients-could-speak-swallow-and-maintain-airway-protection.

[156] Llovet J.M., Kelley R.K., Villanueva A., Singal A.G., Pikarsky E., Roayaie S., Lencioni R., Koike K., Zucman-Rossi J., Finn R.S. (2021). Hepatocellular carcinoma. Nat. Rev. Dis. Primers..

[157] El-Serag H.B. (2012). Epidemiology of viral hepatitis and hepatocellular carcinoma. Gastroenterology.

[158] Verdera H.C., Kuranda K., Mingozzi F. (2020). AAV vector immunogenicity in humans: a long journey to successful gene transfer. Mol. Ther..

[159] Nault J.-C., Datta S., Imbeaud S., Franconi A., Mallet M., Couchy G., Letouzé E., Pilati C., Verret B., Blanc J.-F. (2015). Recurrent AAV2-related insertional mutagenesis in human hepatocellular carcinomas. Nat. Gen..

[160] La Bella T., Imbeaud S., Peneau C., Mami I., Datta S., Bayard Q., Caruso S., Hirsch T.Z., Calderaro J., Morcrette G. (2020). Adeno-associated virus in the liver: natural history and consequences in tumour development. Gut.

[161] Berns K.I., Byrne B.J., Flotte T.R., Gao G., Hauswirth W.W., Herzog R.W., Muzyczka N., VandenDriessche T., Xiao X., Zolotukhin S., Srivastava A. (2015). Adeno-associated virus type 2 and hepatocellular carcinoma?. Hum. Gen. Ther..

[162] Büning H., Schmidt M. (2015). Adeno-associated vector toxicity-to Be or not to Be?. Mol. Ther..

[163] Russell D.W., Grompe M. (2015). Adeno-associated virus finds its disease. Nat. Genet..

[164] Assaf B.T., Whiteley L.O. (2018). Considerations for preclinical safety assessment of adeno-associated virus gene therapy products. Toxicol. Patho..

[165] FDA (2021). https://www.fda.gov/vaccines-blood-biologics/biologics-guidances/cellular-gene-therapy-guidances.

[166] FDA (2021). Food and drug administration (FDA) cellular, tissue, and gene therapies advisory committee (CTGTAC) meeting #70 toxicity risks of adeno-associated virus (AAV) vectors for gene therapy (GT) [Briefing document]. https://www.fda.gov/media/151599/download.

[167] Hüser D., Gogol-Döring A., Chen W., Heilbronn R. (2014). Adeno-associated virus type 2 wild-type and vector-mediated genomic integration profiles of human diploid fibroblasts analyzed by third-generation PacBio DNA sequencing. J. Virol..

[168] Biomarin Pharmaceutical (2021). U.S. FDA placed a clinical hold on BMN 307 pherless phase 1/2 gene therapy study in adults with PKU based on interim pre-clinical study findings [press release]. https://investors.biomarin.com/2021-09-06-U-S-FDA-Placed-a-Clinical-Hold-on-BMN-307-Phearless-Phase-1-2-Gene-Therapy-Study-in-Adults-with-PKU-Based-on-Interim-Pre-clinical-Study-Findings.

